# MitoTracker Deep Red (MTDR) Is a Metabolic Inhibitor for Targeting Mitochondria and Eradicating Cancer Stem Cells (CSCs), With Anti-Tumor and Anti-Metastatic Activity *In Vivo*

**DOI:** 10.3389/fonc.2021.678343

**Published:** 2021-07-30

**Authors:** Camillo Sargiacomo, Sophie Stonehouse, Zahra Moftakhar, Federica Sotgia, Michael P. Lisanti

**Affiliations:** Translational Medicine, School of Science, Engineering and Environment (SEE), University of Salford, Greater Manchester, United Kingdom

**Keywords:** MitoTracker Deep Red (MTDR), near-infrared dyes (NIR), cancer stem-like cells (CSCs), mitochondria, cancer therapy, anti-tumor activity, anti-metastatic activity

## Abstract

MitoTracker Deep Red (MTDR) is a relatively non-toxic, carbocyanine-based, far-red, fluorescent probe that is routinely used to chemically mark and visualize mitochondria in living cells. Previously, we used MTDR at low nano-molar concentrations to stain and metabolically fractionate breast cancer cells into Mito-high and Mito-low cell sub-populations, by flow-cytometry. Functionally, the Mito-high cell population was specifically enriched in cancer stem cell (CSC) activity, i) showing increased levels of ESA cell surface expression and ALDH activity, ii) elevated 3D anchorage-independent growth, iii) larger overall cell size (>12-μm) and iv) Paclitaxel-resistance. The Mito-high cell population also showed enhanced tumor-initiating activity, in an *in vivo* preclinical animal model. Here, we explored the hypothesis that higher nano-molar concentrations of MTDR could also be used to therapeutically target and eradicate CSCs. For this purpose, we employed an ER(+) cell line (MCF7) and two triple negative cell lines (MDA-MB-231 and MDA-MB-468), as model systems. Remarkably, MTDR inhibited 3D mammosphere formation in MCF7 and MDA-MB-468 cells, with an IC-50 between 50 to 100 nM; similar results were obtained in MDA-MB-231 cells. In addition, we now show that MTDR exhibited near complete inhibition of mitochondrial oxygen consumption rates (OCR) and ATP production, in all three breast cancer cell lines tested, at a level of 500 nM. However, basal glycolytic rates in MCF7 and MDA-MB-468 cells remained unaffected at levels of MTDR of up to 1 μM. We conclude that MTDR can be used to specifically target and eradicate CSCs, by selectively interfering with mitochondrial metabolism, by employing nano-molar concentrations of this chemical entity. In further support of this notion, MTDR significantly inhibited tumor growth and prevented metastasis *in vivo*, in a xenograft model employing MDA-MB-231 cells, with little or no toxicity observed. In contrast, Abemaciclib, an FDA-approved CDK4/6 inhibitor, failed to inhibit metastasis. Therefore, in the future, MTDR could be modified and optimized *via* medicinal chemistry, to further increase its potency and efficacy, for its ultimate clinical use in the metabolic targeting of CSCs for their eradication.

## Introduction

Cancer stem-like cells (CSCs) are a relatively small sub-population of tumor cells that share characteristic features with normal adult stem cells and embryonic stem cells (1–4). As such, CSCs are thought to be a ‘primary biological cause’ for i) tumor regeneration and ii) systemic organismal spread, resulting in the clinical features of tumor recurrence and distant metastasis, ultimately driving treatment failure and premature death in cancer patients undergoing chemo- and radio-therapy (1, 2, 4, 5).

Evidence indicates that CSCs also function in tumor initiation, as isolated CSCs experimentally behave as tumor-initiating cells (TICs) in pre-clinical animal models (1, 2). As approximately 90% of all cancer patients die pre-maturely from metastatic disease world-wide (4), there is a great urgency and unmet clinical need, to develop novel therapies for effectively targeting and eradicating CSCs. Most conventional therapies do not target CSCs and often increase the frequency of CSCs, in the primary tumor and at distant sites.

One new approach to the elimination of CSCs has been through the exploitation of cellular metabolism (4). As CSCs are amongst the most energetic cancer cells, new metabolic inhibitors could be employed to induce ATP depletion to “starve” CSCs to death (4–7). So far, we have identified numerous FDA-approved drugs with off-target mitochondrial side effects that have anti-CSC properties and induce ATP depletion, including the antibiotic Doxycycline, which functions as an inhibitor of mitochondrial protein translation (5). Doxycycline, a long-acting Tetracycline analogue, is currently used for treating diverse forms of infections, such as acne, acne rosacea, and malaria prevention, amongst others. In a recent Phase II clinical study, pre-operative oral Doxycycline (200 mg/day for 14 days) reduced the CSC burden in early breast cancer patients between 17.65% and 66.67%, with a near 90% positive response rate (8).

Therefore, it is imperative that we identify other complementary approaches with higher potency, to metabolically starve CSCs by targeting mitochondria and driving ATP depletion. Previous studies have shown that, in general, cyanine dyes accumulate in cells derived from solid tumors, e.g., prostate (9), gastric (10), kidney (11), hepatocytes (12, 13), lung (14) and glioblastoma (15), but not in healthy cells *in vitro* (16–20). More specifically, they observed that cyanine dyes preferentially target mitochondria in cancer cells, by generating a selective chemically-induced cytotoxicity, through redox-based mechanisms (21). In addition, *in vivo* experiments have shown that NIR cyanine derivatives (e.g. IR-780) in general are safe to use, with short-term accumulation and a serum half-life of minutes to hours (22), whereas, in tumors its fluorescent signal persists for days in animals. In addition, they observed that the thiol reactive chloro-methyl moiety (*meso*-chlorine-group) increased IR-780 tumor localization *in vivo*. Therefore, these compounds have been used mainly for theranostic approaches, as well as for photodynamic and photothermal therapy (17, 23–25).

Here, we propose to repurpose the heptamethine cyanine dye MitoTracker Deep Red (MTDR) as a potential therapeutic for targeting mitochondrial metabolism in CSCs. MTDR is currently used as a non-toxic fluorescent chemical probe with a thiol reactive chloromethyl-moiety for visualizing, in the long term, the distribution of mitochondria in living cells and, in the short term, to quantitate mitochondrial potential by FACS or fluorescent microscopy analysis. Recent evidence also indicates that MTDR can also be used as a marker to purify drug-resistant CSC activity by flow-cytometry (26, 27), which was validated by other functional assays, including pre-clinical animal models that documented higher tumor-initiating activity *in vivo* (26).

In the current work, we investigated the anti-cancer properties of MTDR and other NIR dyes, namely HITC, DDI, and IR-780. Interestingly, we found that MTDR, HITC and DDI were all effective inhibitors of anchorage-independent CSC propagation. However, IR-780 had no significant effect in the nanomolar range. In addition, we tested seven Cyanine 5 (Cy5) heptamethine analogs, with different reactive groups, for their ability to inhibit CSC growth. Overall, we observed that Cy5-Azide and Cy5-Alkyne are both effective inhibitors of CSCs, in the nanomolar range.

## Materials and Methods

### Compounds

#### MitoTracker™Deep Red FM

**MTDR** (1-{4-[(chloromethyl)phenyl]methyl}-3,3-dimethyl-2-[5-(1,3,3-trimethyl-1,3-dihydro-2H indol-2-ylidene)penta-1,3-dien-1-yl]-3H-indolium chloride 2-[5-(1-{[4-(chloromethyl)phenyl]methyl}-3,3-dimethyl-1,3-dihydro-2H-indol-2 ylidene) penta-1,3 dien-1-yl]-1,3,3-trimethyl-3H-indolium chloride) was purchased from ThermoFisher (# M22426).

#### Near-Infrared Compounds

HITC iodine (B-1,1’,3,3,3’,3’-Hexamethylindotri-carbocyanine)(# 252034, Merk), DDI iodine (1,1’Diethyl-2-2’-dicarbo-cyanine)(# 392197, Merk) and IR780 iodine (2-[2-[2-Chloro-3-[(1,3-dihydro-3,3-dimethyl-1-propyl-2H-indol 2ylidene)ethylidene]-1-cyclohexen-1-l] ethenyl] -3,3-dimethyl-1 propylindolium iodide) (# 425311, Merk). To prepare a 10 mM stock solution all NIR compounds were first dissolved in PBS, DMEM with 10% FBS, DMEM/F-12. DDI and HITC were dissolved best in water, whereas IR780 in DMEM media (10% FBS). All compounds were left to dissolve for a few hours rolling at room temperature and filtered using a 0.02 µm filter, prior to treating cells. Stock solutions were kept at -20°C.

#### Cy5 Analogs

**Cy5 analogs** were purchased from Lumiprobe (Cyanine5 NHS ester (#13020), Cyanine5 Maleimide (#13080), Cyanine5 Azide (#A3030), Cyanine5 Alkyne (#A30B0), Cyanine5 Hydrazide (#13070), Cyanine5 Amine (#130C0), Cyanine5 Carboxylic Acid (#13090). All Cy5 compounds were dissolved at 10 mM in DMSO and stored at -20°C.

### Cell Lines

Human breast cancer cell lines (MCF7, MDA-MB-231 and MDA-MB-468) were all obtained from the American Type Culture Collection (ATCC).

### 3D-Mammosphere Formation

A single cell suspension was prepared using enzymatic (1x Trypsin-EDTA, Sigma Aldrich, cat. #T3924), and manual disaggregation (25-gauge needle). Five thousand cells were plated with in mammosphere medium (DMEM-F12/B27/20ng/ml EGF/PenStrep), under non-adherent conditions, in six wells plates coated with 2-hydroxyethylmethacrylate (poly-HEMA, Sigma, cat. #P3932). Cells were grown for 5 days and maintained in a humidified incubator at 37°C at an atmospheric pressure in 5% (v/v) carbon dioxide/air. After 5 days, 3D mammospheres with a diameter greater than 50-µm were counted using a microscope, fitted with a graticule eye-piece, and the percentage of cells which formed spheroids was calculated and normalized to one (1 = 100% MFE; mammosphere forming efficiency). Mammosphere assays were performed in triplicate and repeated three times independently.

### Metabolic Flux Analysis on the Total Cell Population

Extracellular acidification rates (ECAR) and oxygen consumption rates (OCR) were analyzed using the Seahorse XFe96 analyzer (Agilent/Seahorse Bioscience, USA). Cells were maintained in DMEM supplemented with 10% FBS (fetal bovine serum), 2 mM GlutaMAX, and 1% Pen- Strep. Twenty-thousand breast cancer cells were seeded per well, into XFe96-well cell culture plates, and incubated at 37°C in a 5% CO_2_ humidified atmosphere. After 24-48 hours, MCF7 cells were washed in pre-warmed XF assay media, as previously described. ECAR and OCR measurements were normalized for cell protein content, by the SRB colorimetric assay. Data sets were analyzed using XFe96 software and Excel software.

### Measuring the Metabolic Effects of MTDR in a CSC-Enriched MCF7 Cell Population

To quantitatively measure the mitochondrial-specific effects of MTDR on a CSC-enriched cell population, we used the Seahorse XFe96 Analyzer to perform the XF Cell Mito Stress Test. Briefly, MCF7 cells were seeded for 3D-mammosphere formation and were grown for 5-days using the standard protocol, but in the absence or presence of MTDR at a low concentration (100 nM), to avoid cell death. Then, the resulting 3D-mammospheres were collected, dissociated into single cells by incubation with trypsin (for 10 minutes at 37°C) and passage through a syringe. The single cell suspension was then passed through a 40-μm strainer, to remove potentially aggregated material and then washed with OCR media. Single cells were counted using Trypan blue to assess their vitality, prior to metabolic analysis. Then, thirty-thousand single cells were dispensed into each well of the 96-well XF microplate pre-coated with Corning™ Cell-Tak Cell and Tissue Adhesive (Catalog No. CB-40240), to ensure rapid adhesion of the cells. Finally, the single cells were briefly centrifuged (200 x g for 1 minute) to further ensure their attachment to the bottom of the wells. Finally, OCR was measured using the Seahorse Metabolic Flux Analyser (XFe96), under standard conditions at 37°C.

### Cell Viability Assays

The effects of MTDR on cell viability were measured by staining cell monolayers with the nuclear fluorescent dye Hoechst 33342, which labels DNA in living cells. Quantitation was performed using a Varioskan LUX multimode microplate reader. The treatment was conducted for 72 hours prior to microplate analysis.

### Fluorescent Microscopy Analysis

Microscopy analysis was performed by analyzing samples in live cell imaging with EVOS imaging platform (ThermoFisher) using the Cy5 channel and bright field.

### Tumor Growth, Metastasis and Embryo Toxicity Assays

Xenograft studies were performed, essentially as previously described (28). According to the French legislation, no ethical approval is needed for scientific experimentation using oviparous embryos (decree n° 2013–118, February 1, 2013; art. R-214–88). Animal studies were performed under animal experimentation permit N° 381029 and B3851610001 to Jean Viallet (INOVOTION). Briefly, fertilized White Leghorn eggs were incubated at 37.5°C with 50% relative humidity for 9 days. At that moment (E9), the chorioallantoic membrane (CAM) was dropped down by drilling a small hole through the eggshell into the air sac, and a 1 cm² window was cut in the eggshell above the CAM. For amplification and grafting of the tumor cells, the MDA-MB-231 tumor cell line was cultivated in DMEM supplemented with 10% FBS and 1% penicillin/streptomycin. On day E9, MDA-MB-231 cells were trypsinized, washed with complete medium and suspended in graft medium. More specifically, we applied Corning^®^ Matrigel^®^ Matrix (Catalogue number: 354230) for tumor cell xenografting. This was performed because it has been demonstrated that the increased viscosity of the cell suspension can prevent the diffusion of cells at the injection site. Briefly, tumor cells are suspended in Matrigel diluted with complete media at 1:1 ratio and then grafted in 50 µl/egg. For the MDA-MB-231 tumor model, an inoculum of 1 x 10^6^ cells in 50µL (i.e., 25 µl Matrigel + 25µl complete culture media) was added onto the CAM of each egg at E9. On day E10, tumors were detectable, and they were then treated daily for 8 days with vehicle alone (1% DMSO in PBS) or with different dosages of MTDR. The therapeutic solution was added dropwise onto the tumor. At day 18 (E18), the upper portion of the CAM was removed from each egg, washed in PBS and then directly transferred to paraformaldehyde (fixation for 48 h) and weighed. For tumor growth assays, at least 10 tumor samples were collected and analyzed per group (n ≥ 10). On day E18, a 1 cm² portion of the lower CAM was collected to evaluate the number of metastatic cells in 7 to 8 samples per group (n ≥ 7). Genomic DNA was extracted from the CAM (commercial kit) and analyzed by qPCR with specific primers for Human Alu sequences. Calculation of Cq for each sample, mean Cq and relative amounts of metastases for each group were directly managed by the Bio-Rad^®^ CFX Maestro software. A one-way ANOVA analysis with post-tests was performed on all the data. To measure embryo tolerability, before each administration, the treatment toxicity was evaluated by scoring the number of dead embryos.

### Statistical Significance

The statistical tests used were the unpaired Student’s t-test and one-way ANOVA. Bar graphs are shown as the average ± SEM (standard error of the mean). A p-value of less than 0.05 was considered statistically significant and is indicated by asterisks: *p < 0.05, **p < 0.01, ***p < 0.001 and ****p < 0.0001. ns, not significant.

### Software

All statistical analysis was performed using GraphPad version 8. All compounds chemical structures were drawn by using ChemDraw 18.

## Results

Here, we directly assessed the suitability of using MitoTracker Deep Red (MTDR), a well-known mitochondrial fluorescent probe, for targeting mitochondria and effectively inhibiting the propagation of breast CSCs. For this purpose, we employed three model cell lines of breast cancer: MCF7, MDA-MB-231, MDA-MB-468, as well as an hTERT-BJ1 skin fibroblasts cell line.

MCF7 is an ER (+) breast cancer cell line, while MDA-MB-231 and MDA-MB-468 are both triple negative [ER (-), PR (-), HER2 (-)] cell lines. In this context, we assessed the targeted effects of MTDR on 3D CSC propagation and overall metabolic rates in 2D monolayer cultures.

**Figure 1:**
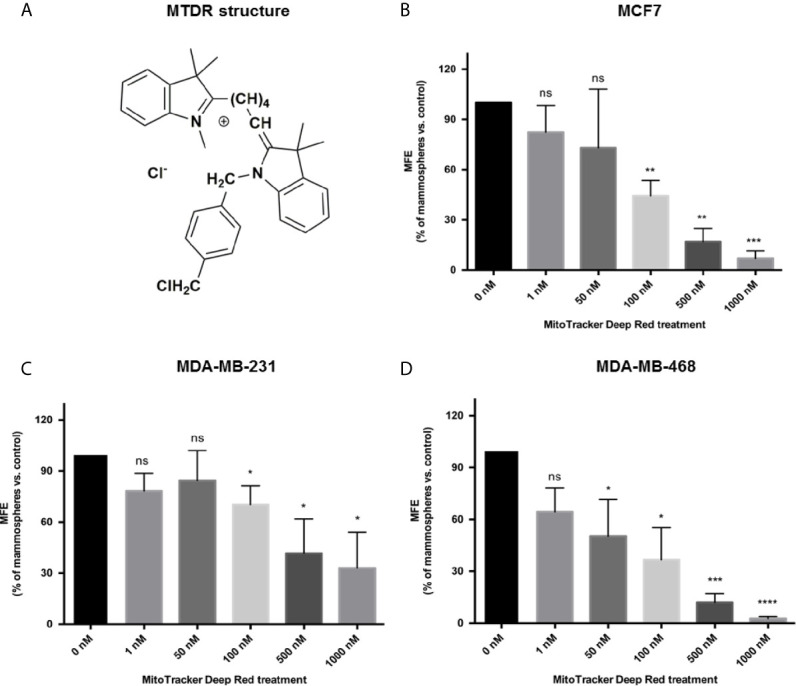
MTDR inhibits mammosphere formation in MCF7, MDA-MB-231 and MDA-MB-468 cells. **(A)** *Structure of MitoTracker Deep Red FM (MTDR).* MTDR is a far-red fluorescent dye that stains active mitochondria. Note that MTDR is a lipophilic cation, which is a chemical characteristic that results its high-efficiency targeting to mitochondria. **(B)** *Effects of MTDR on 3D mammosphere formation in MCF7 cells.* Note that MTDR inhibits 3D anchorage-independent growth in MCF7 cells, with an IC-50 of <100 nM. **(C)** *Effects of MTDR on 3D mammosphere formation in MDA-MB-231 cells.* Note that MTDR inhibits 3D anchorage-independent growth in MDA-MB-231 cells, with an IC-50 between 100 and 500 nM. **(D)** *Effects of MTDR on 3D mammosphere formation in MDA-MB-468 cells.* Note that MTDR inhibits 3D anchorage-independent growth in MDA-MB-468 cells, with an IC-50 between 50 and 100 nM. The statistical test used was a one-tail unpaired t-test.

The structure of MTDR is shown in **Figure 1A**.

### MTDR Inhibits the 3D Anchorage-Independent Propagation of CSCs

In order to assess the effects of MTDR on CSC propagation, we used the mammosphere assay as a functional readout of “stemness” and 3D anchorage-independent growth. As CSCs are highly-resistant to many types of cell stress, they can undergo anchorage-independent propagation, under low-attachment conditions. Ultimately, this results in the generation of >50 μm sized 3D spheroid-like structures. These “mammospheres” are highly enriched in CSCs and progenitor-like cells, and morphologically resemble the morula stage of embryonic development, a solid “ball” of cells without a hollow lumen. Under these culture conditions of non-attachment, the majority of epithelioid cancer cells die, *via* an unusual form of apoptosis, known as “anoikis” (29).

Remarkably, each single 3D mammosphere is constructed from the anchorage-independent clonal propagation of an individual CSC, and does not involve the process of self-aggregation, under these limiting dilution conditions. As a consequence, the growth of 3D spheroids provides functional culture conditions to select for a population of epithelioid CSCs, with EMT properties. As such this provides an ideal assay for identifying small molecules that can target the anchorage-independent growth of CSCs.

**Figure 1B** shows that MTDR potently inhibits 3D spheroid formation in MCF7 cells, with an IC-50 of less than 100 nM. Similarly, MTDR also inhibited the anchorage-independent growth of MDA-MB-231 cells, in the range of 100 to 500 nM (**Figure 1C**). Finally, MTDR inhibited 3D sphere formation in MDA-MB-468 cells, with an IC-50 of approximately 50 nM (**Figure 1D**). Therefore, MTDR is clearly effective in targeting CSCs, in both ER (+) and triple-negative breast cancer-derived cell lines, in the nano-molar range.

### MTDR Specifically Inhibits Mitochondrial Metabolism

To validate the idea that MTDR was specifically targeting mitochondrial metabolism, we next subjected monolayer cultures to metabolic flux analysis, using the Seahorse XFe96 analyzer.

**Figure 2:**
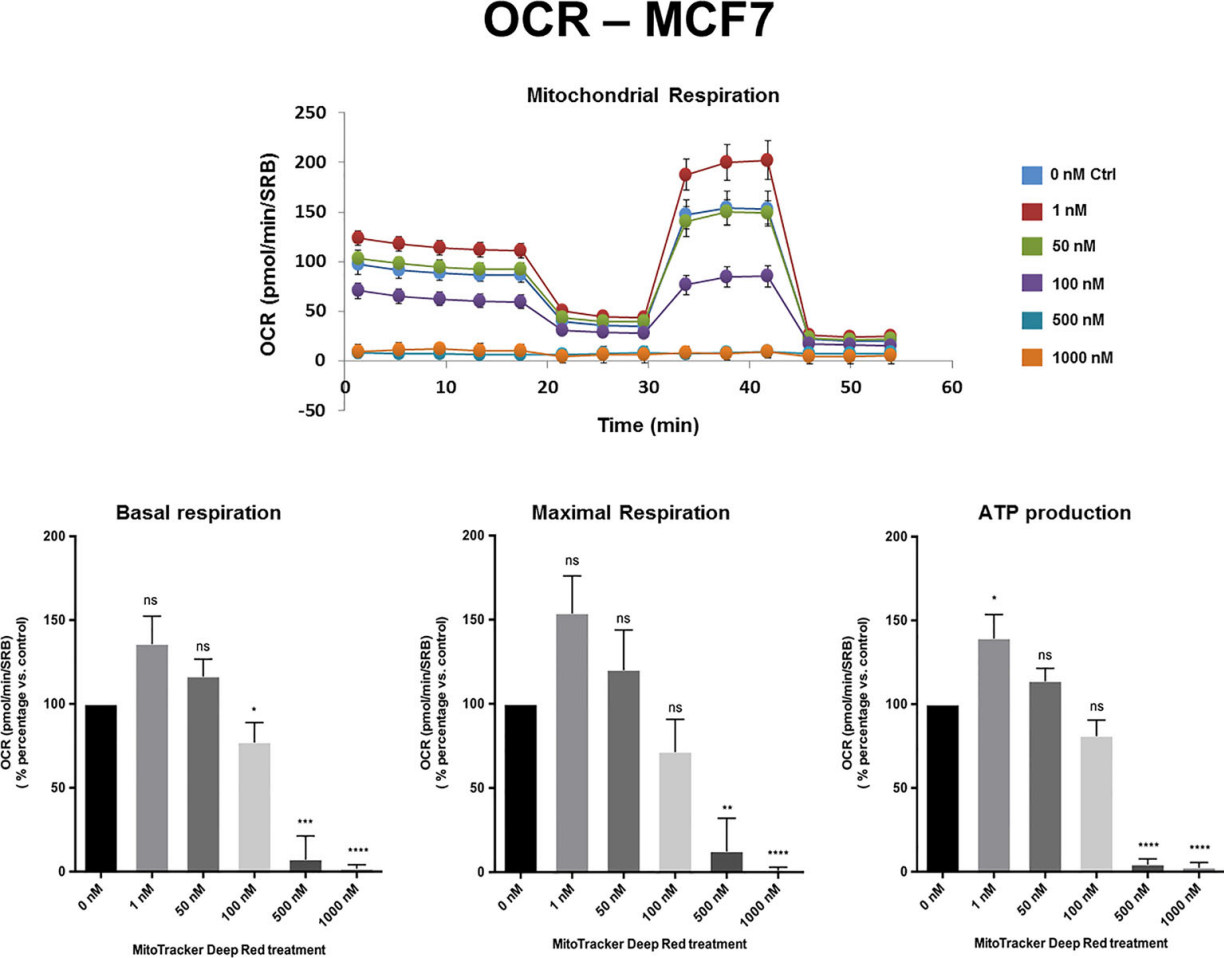
MTDR potently inhibits the mitochondrial oxygen consumption rate (OCR) in MCF7 cells. A representative Seahorse tracing is shown, with bar graphs highlighting the quantitative, dose-dependent effects of MTDR on basal respiration, maximal respiration and ATP production. Note that MTDR treatment exhibits near complete inhibition of OCR in MCF7 cells at 500 nM. The statistical test used was a one-tail unpaired t-test.

**Figure 3:**
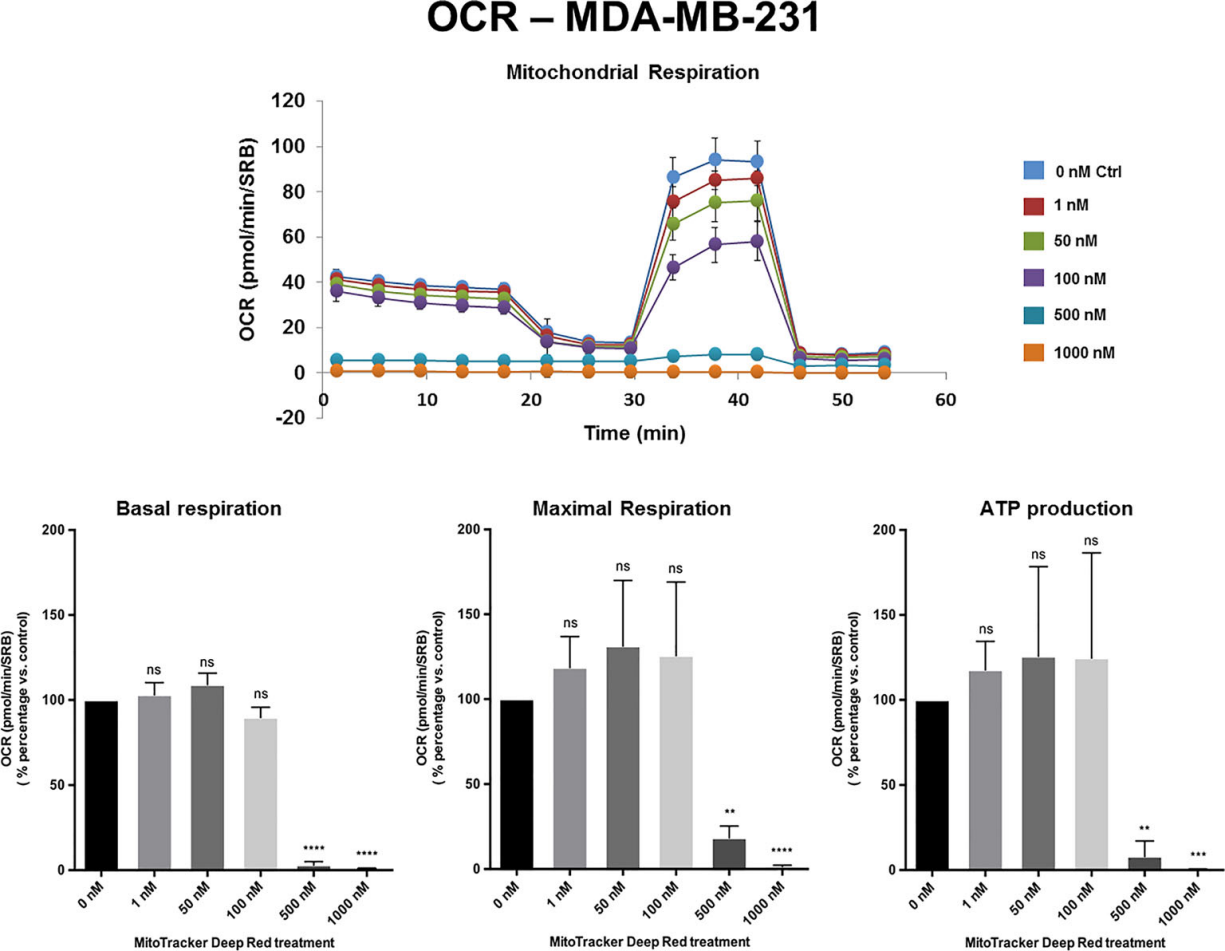
MTDR potently inhibits the mitochondrial OCR in MDA-MB-231 cells. A representative Seahorse tracing is shown, with bar graphs highlighting the quantitative, dose-dependent effects of MTDR on basal respiration, maximal respiration and ATP production. Note that MTDR treatment exhibits near complete inhibition of OCR in MDA-MB-231 cells at 500 nM. The statistical test used was a one-tail unpaired t-test.

**Figures 2****,****3** and **Supplemental Figure S1** show the effects of MTDR on the mitochondrial oxygen consumption rate (OCR) in MCF7, MDA-MB-231 and MDA-MB-468 monolayer cells, respectively. In all three cell lines, MTDR treatment induced near complete inhibition of mitochondrial basal and maximal respiration and ATP production, starting at a concentration of 500 nM.

**Figure 4:**
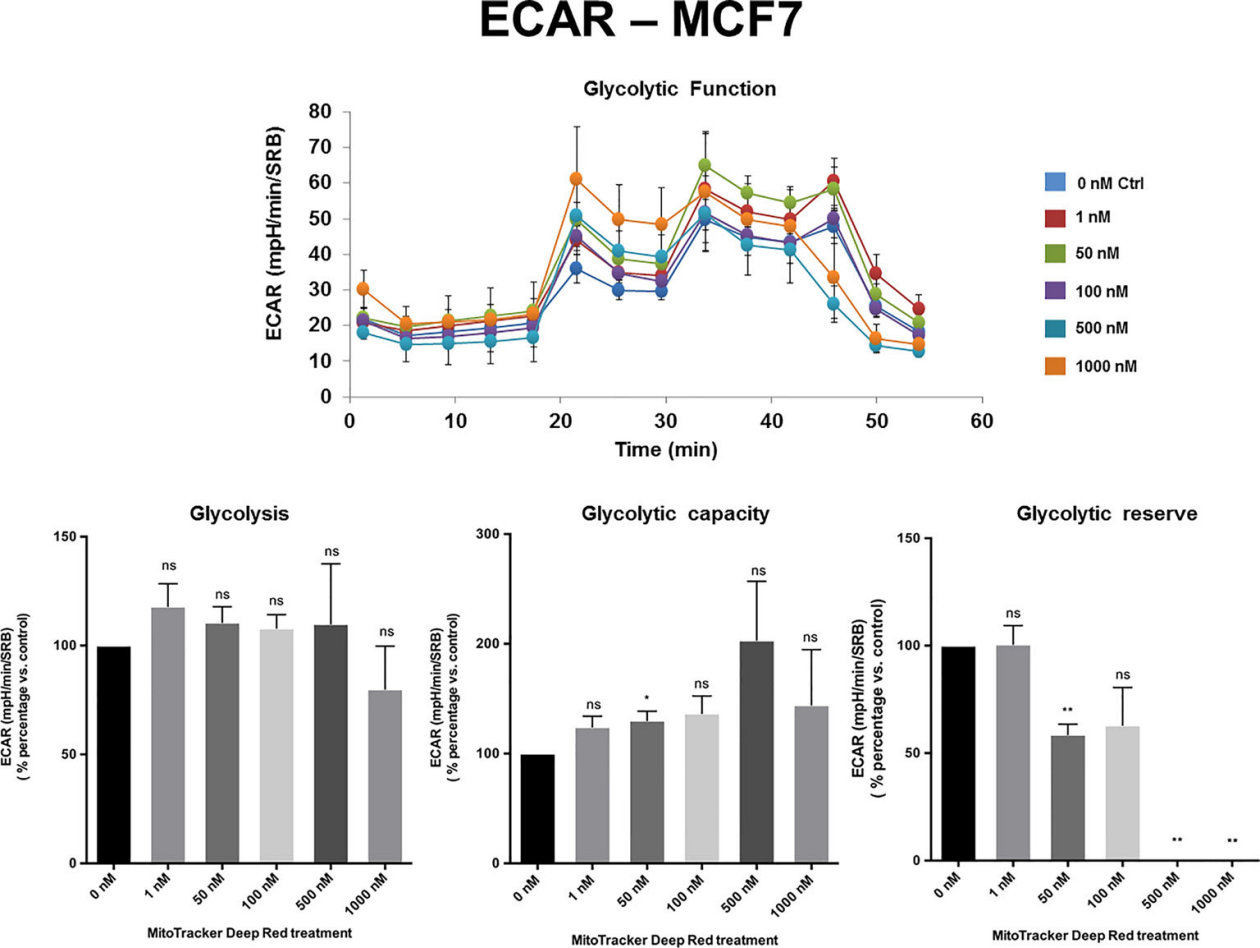
MTDR has no effect on glycolysis in MCF7 cells. A representative Seahorse tracing is shown, with bar graphs highlighting the quantitative, dose-dependent effects of MTDR on glycolysis, glycolytic capacity and glycolytic reserve. Note that MTDR has no significant effect on glycolysis, at concentrations up to 1 μM. The statistical test used was a one-tail unpaired t-test.

The extracellular acidification rate (ECAR), a measure of glycolytic function, remained largely unchanged in MCF7 and MDA-MB-468 cell monolayers, at levels of MTDR of up to 1 µM. Similarly, in MDA-MB-231 cells, the levels of glycolysis remained unchanged from 1 nM to 100 nM MTDR, but glycolysis was decreased at levels of 500 to 1,000 nM, especially for glycolytic reserve (**Figures 4** **,** **5** and **Supplemental Figure S2**). Therefore, high nano-molar concentrations of MTDR, of 500 nM or greater, preferentially affected mitochondrial metabolism in all three breast cancer cell lines tested.

**Figure 5:**
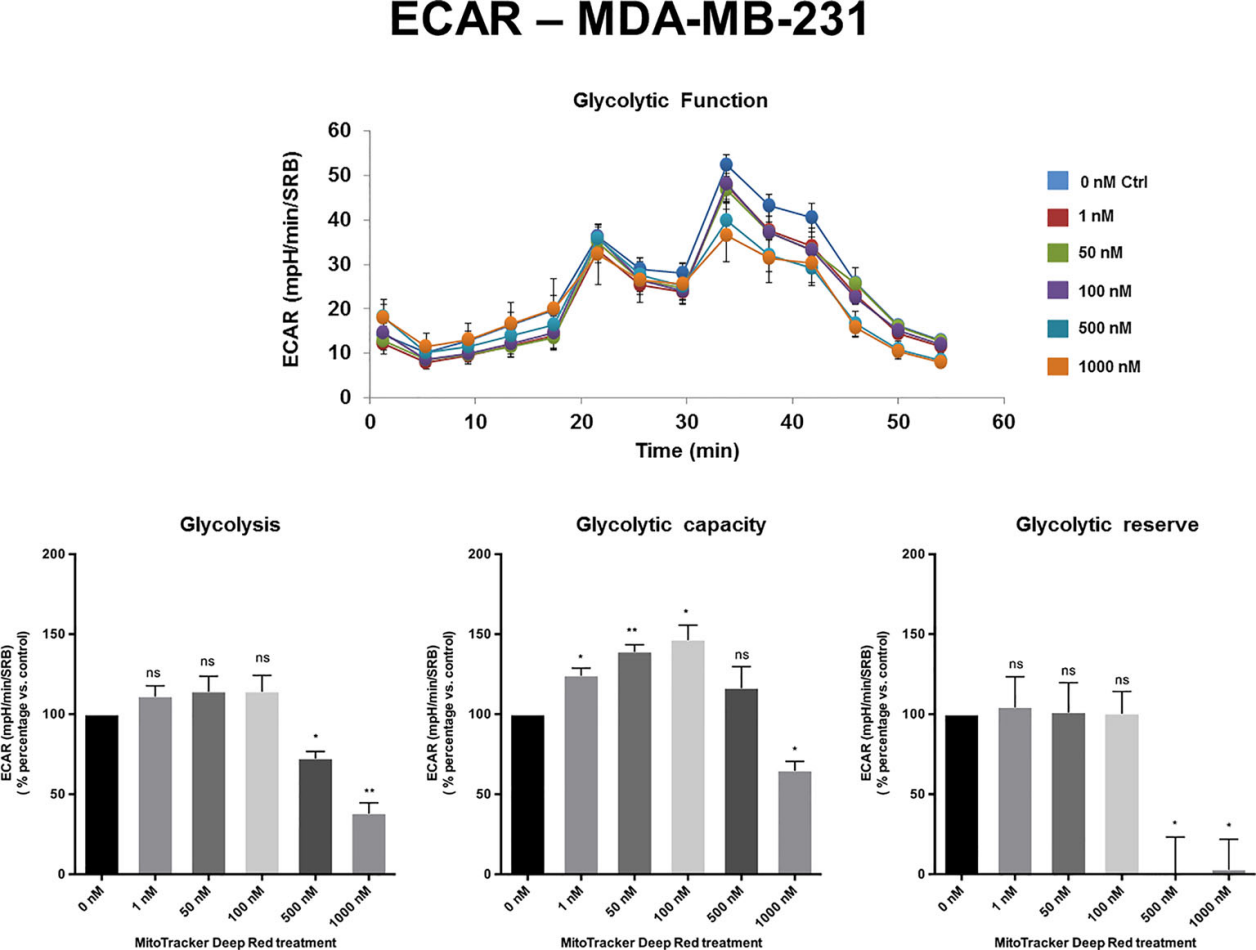
MTDR has minor effects on glycolysis in MDA-MB-231 cells. A representative Seahorse tracing is shown, with bar graphs highlighting the quantitative, dose-dependent effects of MTDR on glycolysis, glycolytic capacity and glycolytic reserve. Note that MTDR has no significant effect on glycolysis, at concentrations up to 100 nM. Mild to moderate inhibition of glycolysis was only observed, starting at 500 nM. The statistical test used was a one-tail unpaired t-test.

### Validating the Metabolic Effects of MTDR in a CSC-Enriched MCF7 Cell Population

To further evaluate the metabolic effects of MTDR, we measured the mitochondrial-specific effects of MTDR on a CSC-enriched MCF7 cell population, using the Seahorse XFe96 Analyzer to perform the XF Cell Mito Stress Test.

Briefly, MCF7 cells were seeded for 3D-mammosphere formation and were grown for 5-days using the standard protocol, but in the absence or presence of MTDR at a lower concentration (100 nM), to avoid cell death. Then, the resulting 3D-mammospheres were collected, dissociated into single cells and subjected to OCR analysis.

**Figure 6:**
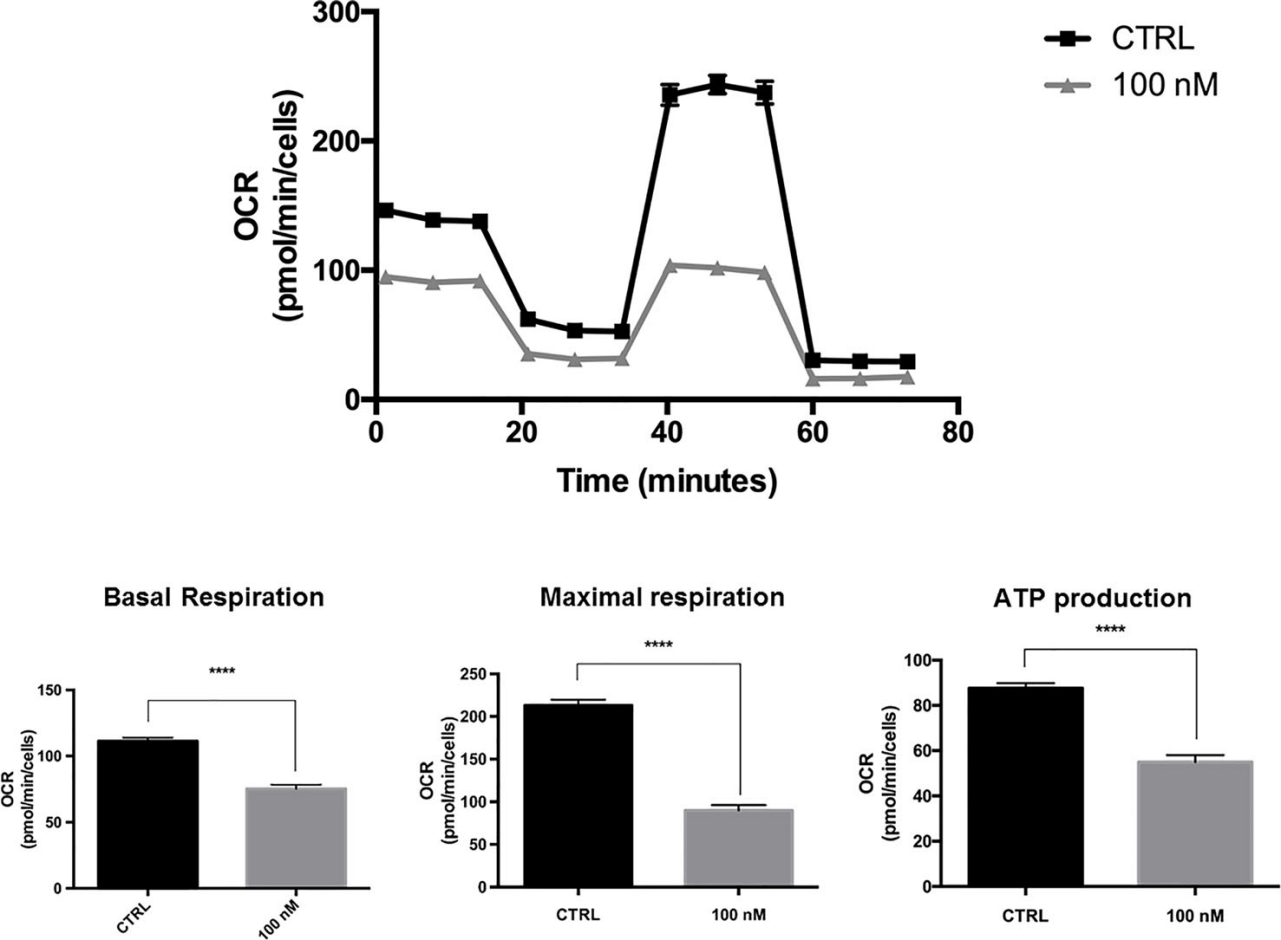
MTDR potently inhibits the mitochondrial OCR in a CSC-enriched MCF7 cell population. A representative Seahorse tracing is shown, with bar graphs highlighting the quantitative effects of MTDR on basal respiration, maximal respiration and ATP production. Note that MTDR treatment exhibits inhibition of OCR at 100 nM. Briefly, MCF7 cells were seeded for 3D-mammosphere formation and were grown for 5-days using the standard protocol, but in the absence or presence of MTDR at a lower concentration (100 nM), to avoid cell death. Then, the resulting 3D-mammospheres were collected, dissociated into single cells and subjected to OCR analysis. Under these conditions, mitochondrial ATP production was reduced by nearly 50%. The statistical test used was a one-tail unpaired t-test.

**Figure 6** shows that at a concentration as low as 100 nM, MDTR significantly inhibited OCR in this CSC-enriched MCF7 cell population. Under these conditions, mitochondrial ATP production was reduced by nearly 50%.

### MTDR Preferentially and Selectively Targets Cancer Cells

To examine the selectivity of MTDR for the preferential targeting of cancer cells, we used a Hoechst-based viability assay. Briefly, MCF7, MDA-MB-231 and MDA-MB-468 cell monolayers were all treated with MTDR, at concentrations ranging from 1 nM to 1 μM, for a period of 72 hours and their viability was assessed using Hoechst 33342, a nuclear dye that stains DNA in live cells. The viability of normal human fibroblasts (hTERT-BJ1) treated with MTDR was also assessed in parallel. Quantitation was performed with a plate-reader.

**Figure 7:**
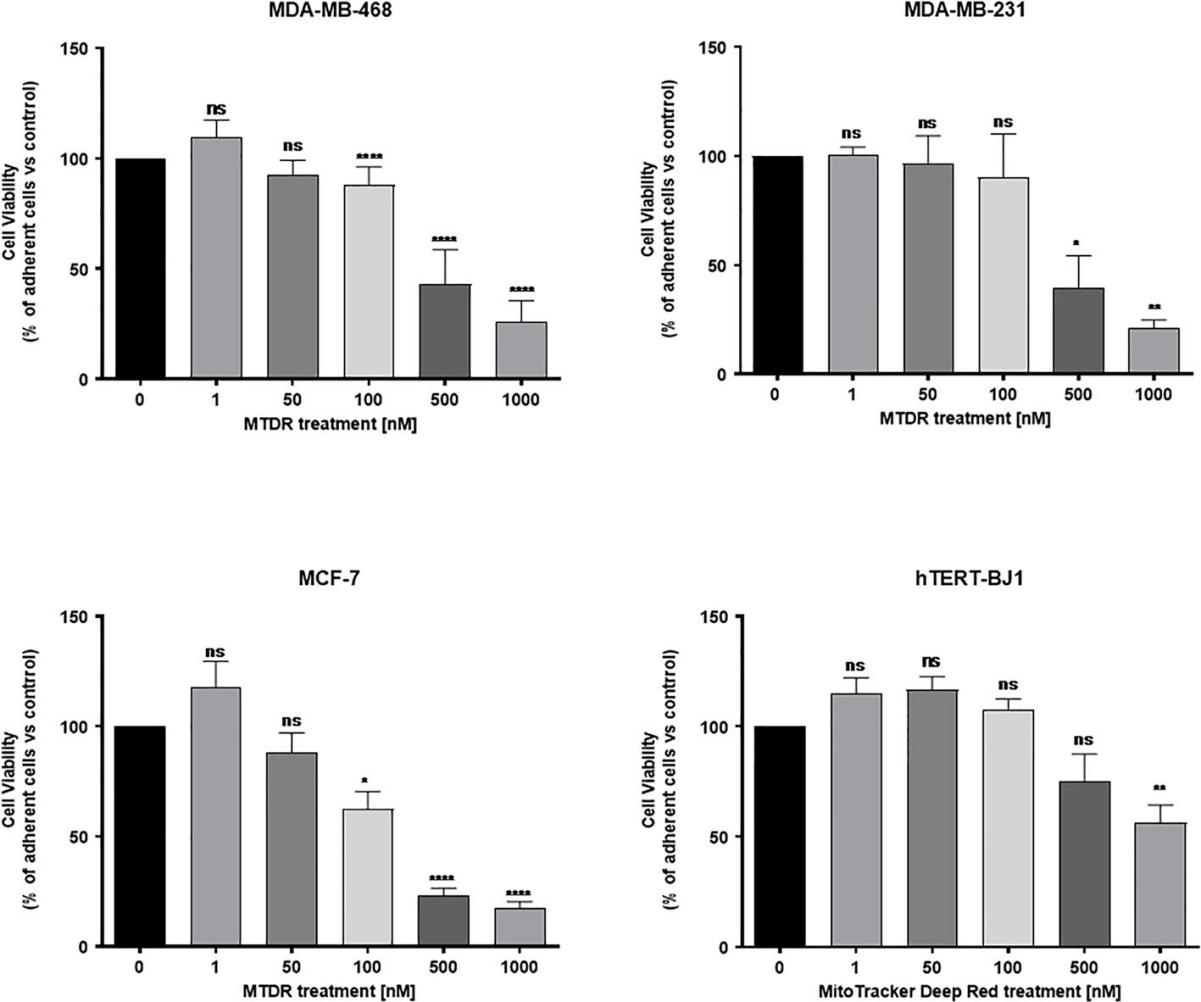
MTDR preferentially and selectively targets cancer cells. MCF7, MDA-MB-231 and MDA-MB-468 cell monolayers were all treated with MTDR for a period of 72 hours and viability was assessed using Hoechst 33342, a nuclear dye that stains DNA in live cells. Effects of MTDR on the viability in normal human fibroblasts (hTERT-BJ1) were assessed in parallel. Note that MTDR effectively killed MCF7, MDA-MB-231 and MDA-MB-468 cells, but had little or no effect on normal cell viability (hTERT-BJ1), in the same concentration range. Therefore, MTDR was more potent and selective for the targeting of breast cancer cells, relative to normal fibroblasts. The statistical test used was a one-tail unpaired t-test.

**Figure 7** shows that MTDR more effectively killed MCF7, MDA-MB-231 and MDA-MB-468, but was less effective on hTERT-BJ1 (a non-transformed fibroblast cell line). Therefore, MTDR was more potent and selective for the targeting of breast cancer cells, with little or no effect on normal fibroblast viability.

### MCF7 Treatment With Other Near-Infrared (NIR) Compounds

**Figure 8:**
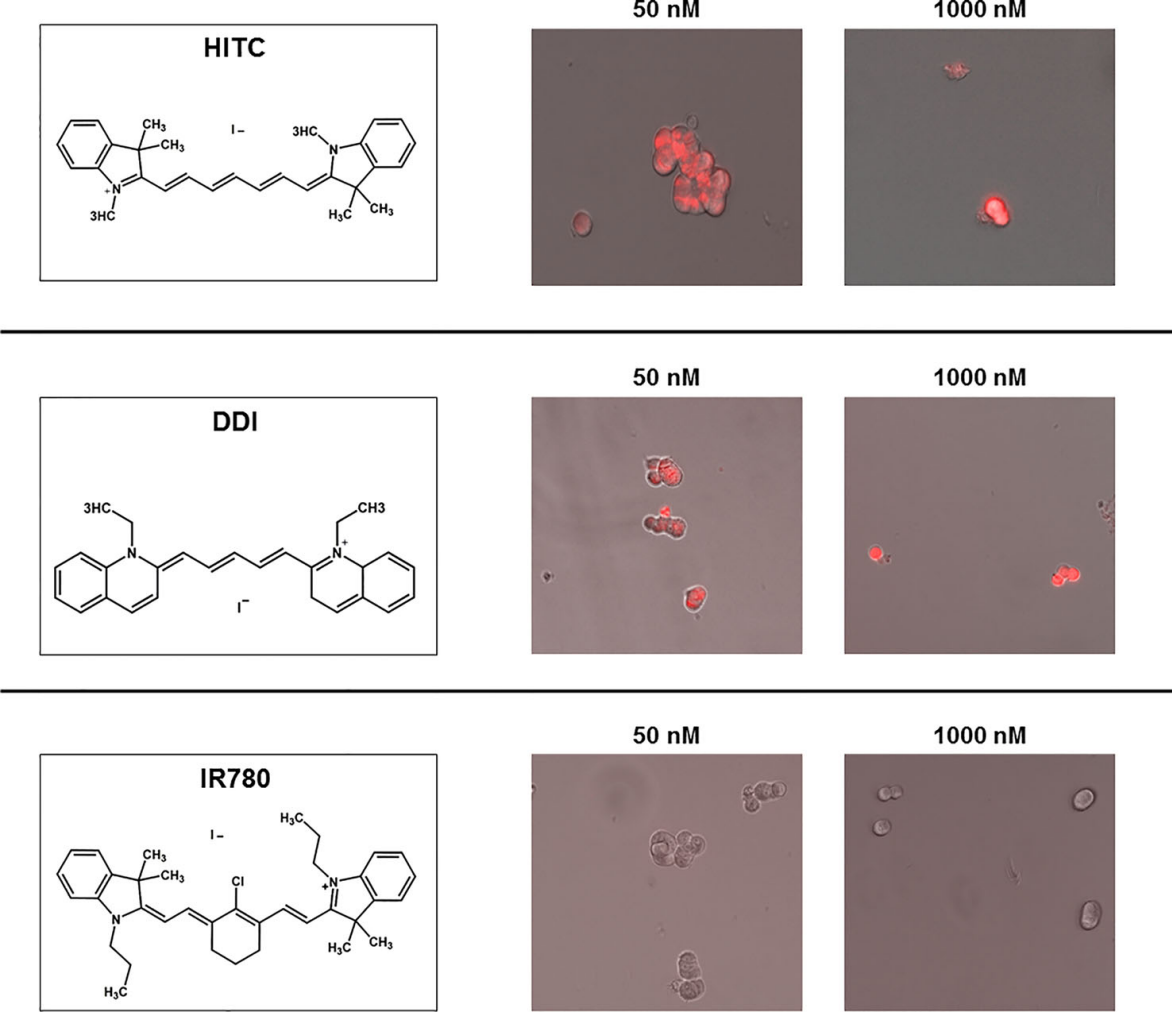
Structure and uptake of NIR compounds in MCF7 cells. (Left). Near-infrared (NIR) compounds. Chemical structures of HITC (B-1,1’,3,3,3’,3’- Hexamethylindotricarbocyanine iodide), DDI (1,1’Diethyl-2-2’-dicarboccyanine iodide) and IR780 (2-[2-[2-Chloro-3-[(1,3-dihydro-3,3-dimethyl-1-propyl-2H-indol-2- ylidene)ethylidene]-1-cyclohexen-1-l]ethenyl]-3,3-dimethyl-1-propylindolium iodide). NIR compounds present a characteristic peak fluorescent emission in the NIR light spectrum. (Middle and Right). MCF7 mammosphere cellular uptake. Treatment of mammospheres using 50 nM and 1000 nM concentrations of HITC, DDI and IR780 for five days. Note that HITC and DDI is localized within the cell and was observed with the Cy5 channel (near infrared signal), whereas IR780 cellular uptake was not detectable, even at higher concentrations.

**Figure 9:**
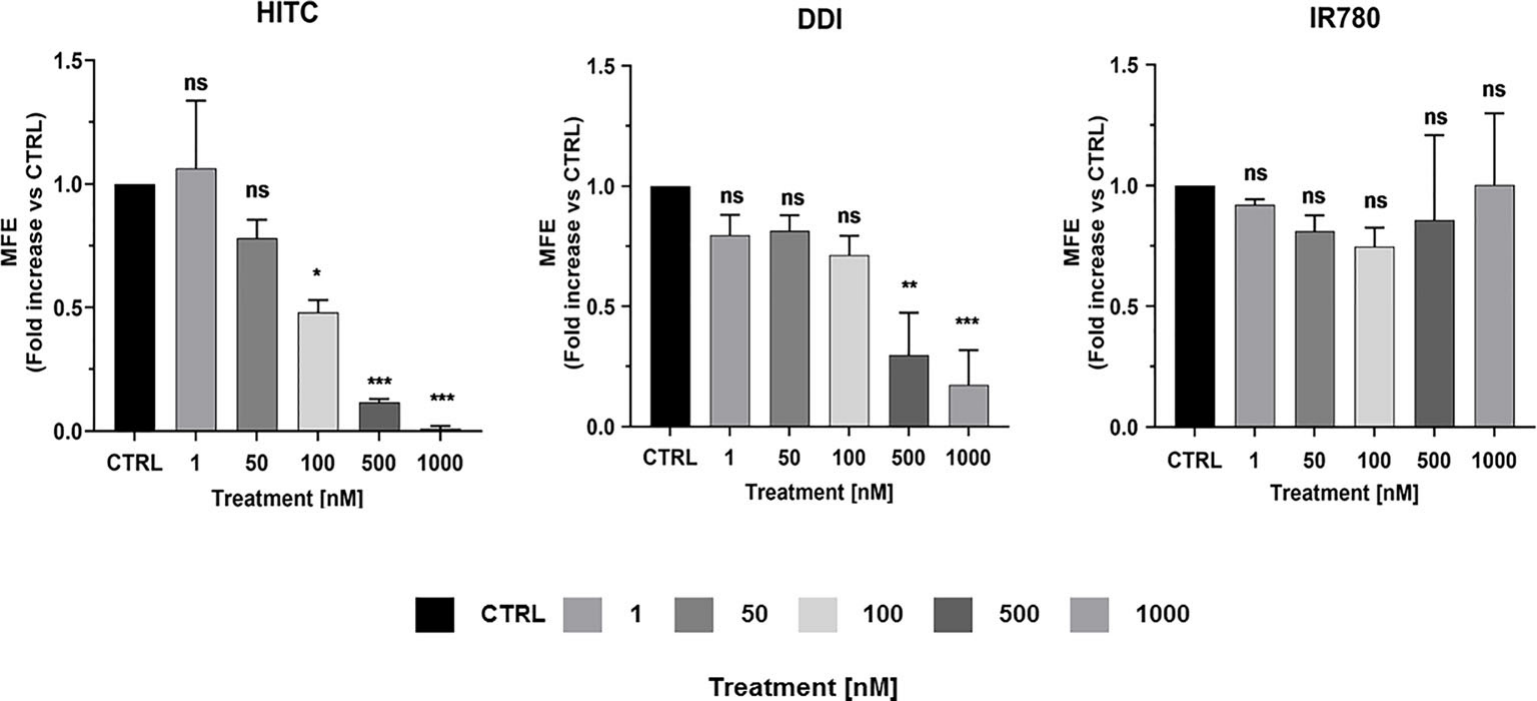
HITC and DDI behave as cancer stem cell inhibitors. Non-adherent MCF7 cells were treated using different drug concentrations of HITC, DDI and IR780 (1, 50, 100, 500, 1,000 nM) for five days and then mammospheres were counted. Data are expressed as fold increase Vs control. Statistical analysis was conducted using one-way ANOVA.

To evaluate the anti-cancer properties other carbocyanine dyes with similar spectral emission as MTDR, we next selected three compounds with a spectral emission in the NIR range (**Figures 8** and **9**) and performed the MCF7 3D-mammosphere assay to assess their effect on CSC propagation. Namely, HITC, DDI and IR-780 were all tested, using the same nanomolar concentration range used for MTDR.

**Figure 9** illustrates that both HITC and DDI significantly inhibited CSC propagation, between 100 and 1,000 nM. In contrast, IR-780, was not effective. In support of these findings, **Figure 8** shows that only HITC and DDI were efficiently incorporated into 3D-mammospheres, while IR780 was not taken up by MCF7 CSCs, at concentrations in the nano-molar range.

To examine the effects of HITC and DDI on mitochondrial respiration and aerobic glycolysis, we treated adherent MCF7 and then measured their OCR and ECAR. **Supplemental Figure S3** shows that basal and maximal OCR, as well as ATP production levels, were both significantly inhibited, as compared to vehicle-alone control cells. In contrast, ECAR levels were increased significantly, at 500 and 1,000 nM (**Supplemental Figure S4**). Surprisingly, DDI did not affect OCR and ECAR in MCF7 cells (**Supplemental Figures S5**, **S6**).

In summary, our results indicate that HITC specifically targets mitochondrial metabolism and inhibits 3D-mammosphere formation. In contrast, DDI also inhibits 3D-mammosphere formation, but by a mitochondrial-independent mechanism. Finally, IR780 did not inhibit CSC propagation in the nanomolar range.

### MCF7 Treatment With Cyanine 5 (Cy5) Analogs

**Figure 10:**
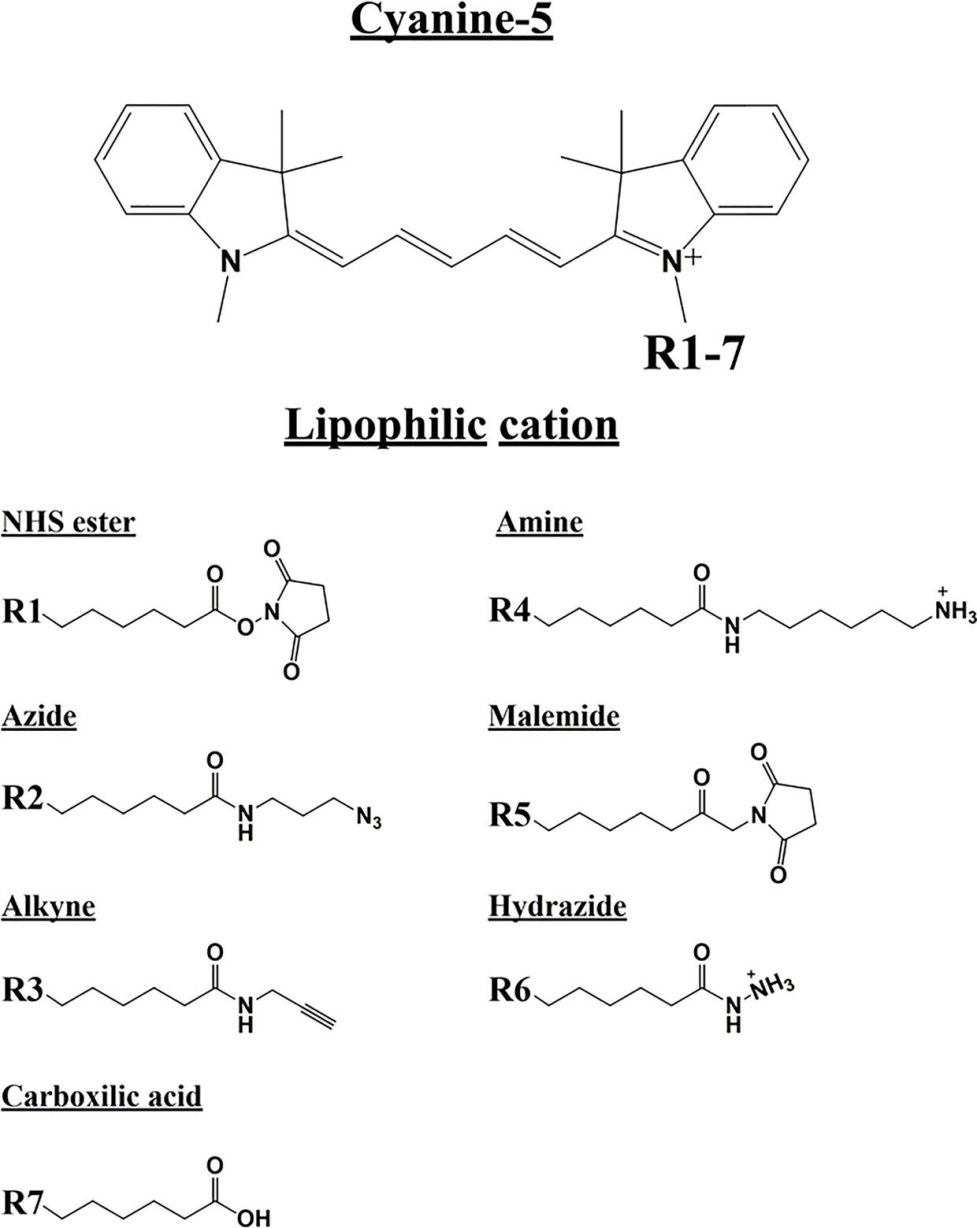
Cyanine 5 structure and analogue structures. Upper: The chemical structure of cyanine 5 compounds is characterized by a polymethine bridge in between the two nitrogen atoms. The positive charge (+) is delocalized within the scaffold on one of the two amine groups (N+). The amine group can be used to attach several different side chains (R1-7). Lower: Below are different Cyanine 5 analogue with their side chain structure highlighted.

To assess the possible anti-cancer properties of Cy5 lipophilic fluorophores, we next tested the potential inhibitory activity of seven commercially available Cy5 analogs (**Figure 10**).

**Figure 11:**
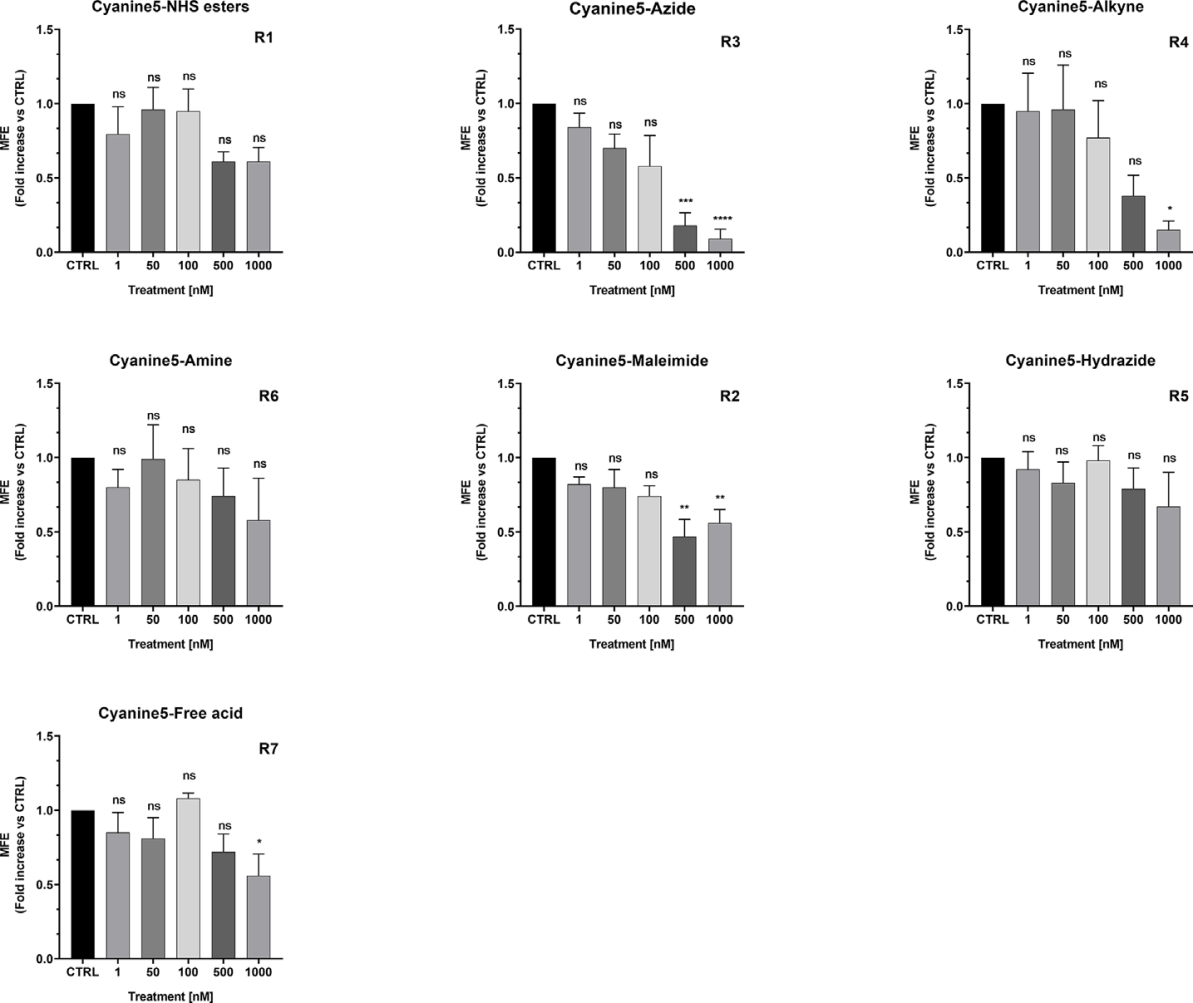
Effects of Cyanine 5 on 3D mammosphere formation in MCF7 cells. MCF7 mammospheres cells were treated with different concentrations of each compound (1, 50, 100, 500 and 1,000 nM) for five days. Mammospheres >50-µm were counted manually using a bright field microscope (n = 4). Data is expressed as fold increase Vs control. Statistical analysis was conducted using one-way ANOVA.

We found that Cy5-Azide and Cy5-Alkyne analogs were the only two compounds out of seven, to significantly inhibit MCF7 3D-mammosphere formation, between 500 nM to 1,000 nM (**Figure 11**). However, all the Cy5 analogs were internalized by mammospheres, at low nanomolar concentrations (50 nM), independently from their anti-CSC effects, and suggesting that Cy5 retention lasts for days in CSCs (**Supplemental Figure S7**).

Furthermore, we found that both carbocyanine compounds (Cy5-Azide and Cy5-Alkyne) are mitochondrial OXPHOS inhibitors that act, in the range of 500 nM and 1000 nM, and they induce glycolysis to compensate for mitochondrial ATP depletion (data not shown).

### MTDR Potently Inhibits Cancer Cell Tumor Growth and Metastasis *In Vivo*

To determine the potential therapeutic effects of MTDR *in vivo*, we next used a human breast cancer cell line, namely MDA-MB-231 cells, and the well-known chorio-allantoic membrane (CAM) assay in chicken eggs, to evaluate the effects of MTDR on tumor growth and metastasis (28, 30).

More specifically, an inoculum of 1 x 10^6^ MDA-MB-231 cells was added on top of the Upper CAM of each egg (day E9). On day E10, tumors were detectable and they were then treated daily for 8 days with vehicle alone (1% DMSO in PBS) or MTDR. After 8 days of MTDR administration, on day E18 all tumors were weighed, and the Lower CAM was collected to evaluate the number of metastatic cells, as analyzed by qPCR with specific primers for Human Alu sequences.

**Figure 12:**
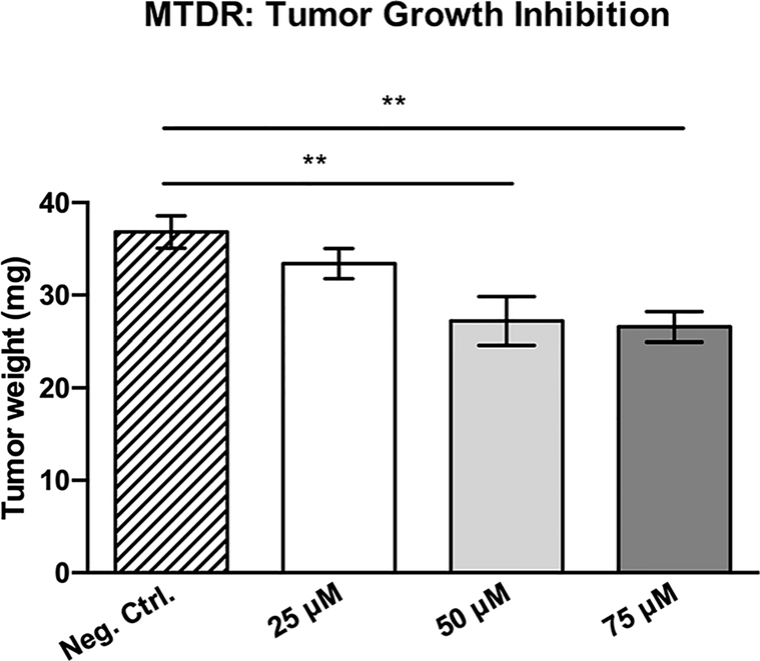
MTDR significantly inhibits tumor growth. MDA-MB-231 cells and the well-established chorio-allantoic membrane (CAM) assay in chicken eggs were used to quantitatively measure tumor growth. See Materials & Methods for specific details. After 8 days of MTDR administration, on day E18 all tumors were weighed. Note that MTDR significantly inhibited tumor growth, by up to nearly 30%, at a concentration of 75 μM (n ≥ 15). Averages are shown ± SEM. **p < 0.01; *p < 0.05. Statistical analysis was conducted using ANOVA.

**Figure 13:**
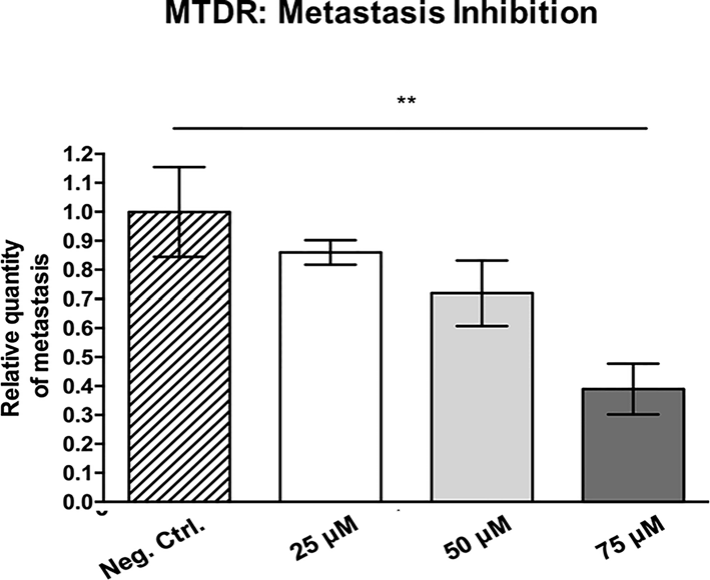
MTDR preferentially targets and prevent cancer cell metastasis. MDA-MB-231 cells and the well-established chorio-allantoic membrane (CAM) assay in chicken eggs were used to quantitatively measure spontaneous tumor cell metastasis. See Materials & Methods for specific details. After 8 days of drug administration, the Lower CAM was collected to evaluate the number of metastatic cells, as analyzed by qPCR with specific primers for Human Alu sequences. Note that MTDR showed significant effects on MDA-MB-231 metastasis, with an inhibition of >60%, at a concentration of 75 μM (n ≥ 7). Averages are shown ± SEM. **p < 0.01. Statistical analysis was conducted using ANOVA.

**Figure 12** shows the effects of MTDR on MDA-MB-231 tumor growth. Note that MTDR showed a significant effect on tumor growth, with inhibition of up to nearly 30%, as a result of the 8-day period of drug administration. In addition, MTDR showed significant effects on MDA-MB-231 cancer cell metastasis. **Figure 13** illustrates that MTDR inhibited metastasis (by >60%) at the same concentrations tested. Interestingly, the effects of MTDR on metastasis were significantly more pronounced.

**Figure 14:**
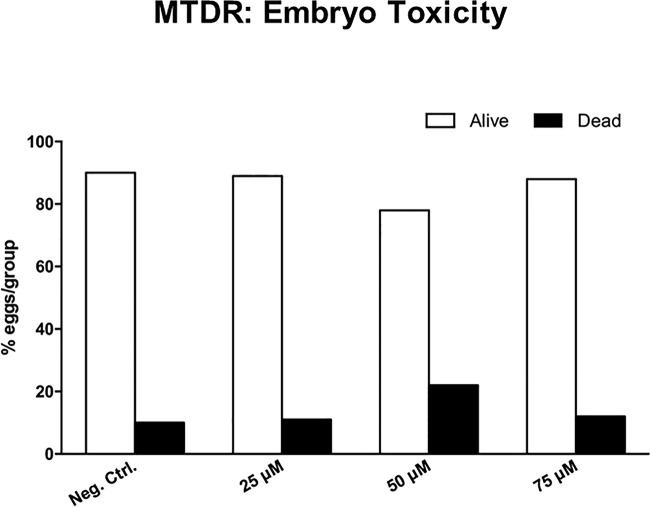
Chick embryo toxicity of MTDR. Note that MTDR was non-toxic, as compared to the vehicle alone control (n ≥ 17), over a dose range of 25, 50, and 75 μM.

Surprisingly, little or no embryo toxicity was observed for MTDR (**Figure 14**). Therefore, we conclude that MTDR can be further developed as an anti-cancer agent, for inhibiting both tumor growth and metastasis, without showing significant toxicity.

### Abemaciclib, a CDK4/6 Inhibitor, Does Not Prevent Metastasis *In Vivo*

For comparison purposes, in parallel, we also assessed the functional activity of an FDA-approved CDK4/6 inhibitor, namely Abemaciclib, which was developed by Eli Lilly. Abemaciclib was first approved in 2017, with breakthrough status, for the treatment of breast cancer patients with advanced or metastatic disease. Results from the MONARCH 3 clinical trial indicated that there was significant clinical benefit for progression-free survival (28.18 versus 14.76 months; hazard ratio [95% confidence interval], 0.540 [0.418–0.698]; p = .000002). As a consequence, Abemaciclib is considered standard-of-care for patients with advanced or metastatic breast cancer (31).

Therefore, here we evaluated the efficacy of Abemaciclib, using the CAM assay, to measure its effects on MDA-MB-231 tumor growth, metastasis and embryo toxicity.

**Figure 15:**
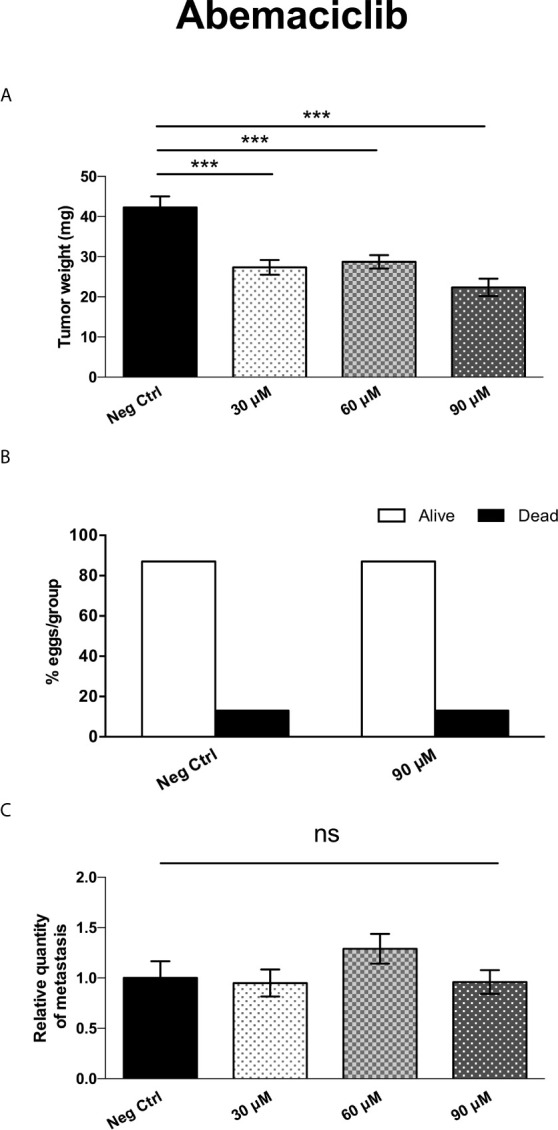
Abemaciclib significantly inhibits tumor growth, without preventing cancer cell metastasis. **(A)** MDA-MB-231 cells and the well-established chorio-allantoic membrane (CAM) assay in chicken eggs were used to quantitatively measure tumor growth. See Materials & Methods for specific details. After 8 days of Abemaciclib administration, on day E18 all tumors were weighed. Note that Abemaciclib significantly inhibited tumor growth, by nearly 50%, at a concentration of 90 μM (n > 11). Averages are shown ± SEM. ***p < 0.001. Statistical analysis was conducted using ANOVA. **(B)** Note that Abemaciclib was non-toxic, as compared to the vehicle alone control (n > 15), up to 90 μM. **(C)** MDA-MB-231 cells and the well-established chorio-allantoic membrane (CAM) assay in chicken eggs were used to quantitatively measure spontaneous tumor cell metastasis. See Materials & Methods for specific details. After 8 days of drug administration, the Lower CAM was collected to evaluate the number of metastatic cells, as analyzed by qPCR with specific primers for Human Alu sequences. Note that Abemaciclib did not show any significant effects on MDA-MB-231 metastasis, at concentrations up to 90 μM (n = 8). Averages are shown + SEM. ns, not significant. Statistical analysis was conducted using ANOVA.

Interestingly, treatment with Abemaciclib (in a similar concentration range as with MTDR) resulted in the significant inhibition of tumor growth by up to 47%, at a 90 μM (**Figure 15A**). Importantly, little or no embryo toxicity was observed in this concentration range (**Figure 15B**). However, Abemaciclib was completely ineffective for the prevention of metastasis (**Figure 15C**).

Thus, as compared with the standard-of-care for advanced breast cancer (Abemaciclib), MTDR was clearly more effective for the prevention of metastatic dissemination, using MDA-MB-231 cells (**Figure 13**).

## Discussion

MTDR is a carbocyanine-based fluorescent probe that chemically behaves as a lipophilic cation. MTDR is functionally localized to mitochondria and is normally used to measure mitochondrial mass in living cells, by fluorescence microscopy and/or FACS analysis. Here, we assessed whether MTDR could be used to target mitochondria in CSCs, to prevent their anchorage-independent propagation. For this purpose, we used three independent breast cancer cell lines, namely MCF7, MDA-MB-231 and MDA-MB-468 cells, representing both ER(+) and triple-negative breast cancer sub-types. Remarkably, we observed that MTDR potently inhibited the 3D propagation CSCs from all three cancer cell lines, using nano-molar concentrations of the near-infrared fluorescent probe. Furthermore, we directly validated that MTDR specifically targeted mitochondrial metabolism and induced ATP depletion, by using the Seahorse XFe96 metabolic flux analyzer. However, we did not see a significant induction of glycolysis by MTDR, in all three cell lines.

Originally, it was thought that MTDR could be used as a probe to measure mitochondrial mass, independently of mitochondrial activity or membrane potential. However, recent experiments directly show that MTDR staining is prevented and/or reduced by treatment with FCCP (carbonyl cyanide 4-(trifluoromethoxy) phenylhydrazone), a potent mitochondrial uncoupling agent (32). Therefore, MTDR may preferentially accumulate in highly-active mitochondria, potentially making it a better therapeutic drug for targeting and inhibiting mitochondrial function.

In addition, we found that other NIR compounds such as HITC and DDI, but not IR780, accumulate in MCF7 cells and inhibit CSC anchorage-independent growth. Our results demonstrated that HITC effectively blocks CSCs growth in a mitochondrial-dependent manner, and induces glycolysis starting at 500 nM. In contrast, we showed that DDI, did not produce any noticeable metabolic effects, but was able to inhibit CSC growth in the nanomolar range in MCF7 cells. Furthermore, we showed that IR-780 was not effective against CSCs growth and was not internalized by tumor cells, at nanomolar concentrations. Thereafter, we compared seven Cy5 carbocyanine analogs with different reactive groups for their ability to inhibit MCF7 CSC growth. Our results showed that all compounds tested were internalized after five days of treatment (**Supplemental Figure S7**). However, only Cy5-Alkyne and Cy5-Azide blocked 3D mammosphere growth and also targeted energized mitochondria in cancer cells (data not shown) within the nanomolar range, which is line with previous studies (33).

We have previously shown that other lipophilic cations, such as derivatives of triphenyl-phosphonium (TPP) (34), can be effectively used to target mitochondria in CSCs, significantly preventing 3D mammosphere formation. However, these TPP derivatives were much less potent, inhibiting 3D spheroid formation in MCF7 cells, with an IC-50 between 500 nM to 5 μM. Therefore, MTDR is clearly more potent than these TPP-derivatives, such as 2,4-dichlorobenzyl-TPP, 1-naphthylmethyl-TPP, 3-methylbenzyl-TPP, 2-chlorobenzyl-TPP, and 2-butene-1,4-bis-TPP. As such, MTDR, HITC, Cy5-Alkyne and Cy5-Azide are clearly more efficacious.

Interestingly, in our work, we identified four carbocyanines that target mitochondria (MTDR, HITC, Cy5-Alkyne and Cy5-Azid) and we found that DDI did not target mitochondria, but it showed the same potency in inhibiting CSC growth. In contrast, IR-780 at nanomolar concentrations, was not internalized and this is in line with previous *in vitro* studies (23). The fact that IR-780 at nanomolar concentrations does not have anti-tumor activity (23, 35), highlights that MTDR, HITC, Cy5-Alkyne and Cy5-Azide are all more effective in eradicating CSCs than IR-780.

In principal, “energized” cancer cell mitochondria present a higher membrane potential (about ~60 mV more negative than in normal cells) (36). Therefore, mitochondria in cancer cells produce more ATP and play a key role for CSC metastatic growth. Indeed, our results are in line with the general theory that CSC strictly depend on mitochondria. We report that in our experiments that glycolysis alone was not sufficient to allow for 3D mammosphere formation, when OCR was targeted by carbocyanines in breast cancer cells. We speculate, as previously demonstrated elsewhere (21), that the lipophilic carbocyanine mechanism of action is through chemical alkylation between electrophilic carbocyanines and nucleophilic atoms within cysteine residues found in mitochondrial proteins (chemical intoxication). In addition, the low pH of the mitochondrial matrix increases chemical alkylation reactions that generate electrophile-protein adducts that are toxic to tumor cells. Therefore, one can speculate that this “non-selective”, chemical intoxication by carbocyanines would presumably allow the avoidance of possible resistance to such broad, but yet organelle-specific, intoxication of cancer cell mitochondria.

Indeed, after observing the Cy5-Alkyne mitochondrial-dependent inhibition of breast CSCs, the same analog was found to show anti-tumor effect, both in *in vitro* and *in vivo*, in the nanomolar range (33), further confirming our findings. Interestingly, Cy5-NHS was found to successfully deliver nano-particles and other compounds such as doxorubicin to mitochondria, and to retain their mitochondrial targeting of tumor cells. Furthermore, in other *in vivo* studies, they found that carbocyanines have a strong safety profile, with low toxicity and fast clearance as theranostic agents, and are considered non-toxic agents for cell live imaging applications *in vitro* (17). Furthermore, acute toxicity studies conducted on mice using 100 nM concentrations of IR-780 demonstrated no acute toxicity, and low organ accumulation was observed (25).

Lipophilic carbocyanine uptake has been found to depend on the Organic Anionic Transporter Protein (OATP) family of transporters (37). OATP transporters function as non-selective sodium-independent co-transporter proteins that regulate the flux of ions and small molecules across the plasma membrane of the cell (e.g., bile salts, bilirubin, steroids and thyroid hormones) (38). OATP transporters influx of small molecules is coupled to the passive efflux of intracellular bicarbonate, glutathione and glutathione adducts. Interestingly, the OATP1B3 isoform was previously described to selectively transport Cy5.5, IR-780 as well as Cy5-Alkyne heptamethine carbocyanines both *in vitro* and *in vivo* (37, 39–42). Furthermore, OATPs expression in cells was found to be induced by hypoxia, i.e., lowering oxygen conditions or inducing pseudo-hypoxia *via* DMOG (dimethyloalyglycine) treatment. OATP expression is positively regulated by the transcription factor of hypoxia induction factor 1 (HIF1α). Therefore, cellular hypoxia increases carbocyanine uptake in tumor cells *via* OATP (33, 42), suggesting that hypoxic cancer cells would be more susceptible to carbocyanines, as compared to normoxic cells, and can be preferentially eliminated by using mitochondrially-targeted carbocyanine dyes.

The *de novo* expression of OATP1B3 and 1B1, in general, is considered specific for the liver, but it was found to increase in several tumors. In breast cancer tissue, as well as other cells lines, OATP1B3 transporters are upregulated (38). However, in normal breast tissue, it is absent. Hence, we believe that carbocyanine absorption could depend on OATP functional expression also for breast CSCs. Therefore, in addition to carbocyanine’s chemical electrophilic properties previously discussed, we must consider the key role of OATP in carbocyanines uptake by CSCs. As far as we are aware, OATP expression has not been examined in CSCs and should be further investigated.

Thiol reactive groups are present in both MTDR and IR-780 carbocyanine structures and are thought to react with cysteine residues through *meso*-chlorine motility inside their chemical structure. Nevertheless, we observed that MTDR, compared to IR-780, was more potent in mitochondrial inhibition. A possible explanation is that IR-780 is a Cy7, whereas MTDR is Cy5 member, indicating specific chemical differences between carbocyanine families regarding their potential anti-tumor properties. Nevertheless, Cy7 compounds, such as HITC, goes against such a hypothesis as it blocked mitochondrial respiration in the nanomolar range, and it is a heptamethine without *meso*-chlorin motility. As such, the differences observed between IR-780 and MTDR anti-CSC efficacy may account for other chemical properties. In addition, an FDA-approved carbocyanine Indocyanines green (ICG), which is very similar to IR-780, and is used as an optical marker routinely in the clinic, is not taken up by tumor cells. One possible explanation that was advanced is that ICG is missing a thiol reactive group, which is responsible for IR-780 adduct formation in tumors (22).

In a recent study, human serum albumin (HSA) was found to bind to IR-780 carbocyanines, *via* reactive thiol group to albumin cysteine residues (22), which explains its rapid decrease in the blood (approximately 30 minutes in the serum for IR-780-like dies). They found that IR-780 generated a higher fluorescent signal in the serum compared to ICG (that does not bind to albumin), and they speculate that IR-780-albumin adducts increase the half-life and the tumor uptake capacity of carbocyanines. Interestingly, Usama et al. also reported that IR-780 albumin-adduct uptake by glioblastoma cells was not inhibited by BSP, or increased by DMOG (hypoxia inducer). However, it was abolished only by applying low temperatures (4°C), which strongly suggests an OATP-independent mechanism of uptake for IR-780-albumin adducts.

Overall, in this work, we have shown how the energetic state of malignant cancer cells can be targeted *via* “tumor-seeking” drugs, by taking advantage of the chemical properties of carbocyanine dyes. Hence, we believe that based on our findings, both *in vitro* and *in vivo*, that carbocyanine-induced mitochondrial cytotoxicity should be generally applicable for preventing CSC-driven metastatic growth, and could be used as a preventive treatment against breast cancer relapse, either shortly after or concurrently with chemo- or radiation therapy.

## Conclusions

In conclusion, here we show that MTDR can be used effectively as a metabolic inhibitor to target mitochondrial function and halt CSC propagation, in the nano-molar range. The metabolic effects of MTDR on mitochondrial OCR and ATP production were directly validated, using the Searhorse XFe96 flux analyzer. Therefore, we propose that MTDR, and other related compounds, could be repurposed, as potent and selective anti-cancer agents, to target the CSC population, in a variety of cancer types.

## Data Availability Statement

The original contributions presented in the study are included in the article/**Supplementary Material**. Further inquiries can be directed to the corresponding authors.

## Author Contributions

ML, FS, and CS conceived and initiated this project. Experiments described in this paper were performed by CS, a post-doctoral fellow in the Lisanti/Sotgia laboratory, and SS, a placement student, as well as ZM, a PhD student. ML and FS wrote the first draft of the manuscript, which was then further edited by CS. All authors contributed to the article and approved the submitted version.

## Funding

This work was supported by research grant funding, provided by Lunella Biotech, Inc. The funder, Lunella Biotech, Inc., only provided the necessary monetary resources to carry out the current study.

## Conflict of Interest

ML and FS hold a minority interest in Lunella Biotech, Inc.

The remaining authors declare that the research was conducted in the absence of any commercial or financial relationships that could be construed as a potential conflict of interest.

## Publisher’s Note

All claims expressed in this article are solely those of the authors and do not necessarily represent those of their affiliated organizations, or those of the publisher, the editors and the reviewers. Any product that may be evaluated in this article, or claim that may be made by its manufacturer, is not guaranteed or endorsed by the publisher.

## Supplementary Material

Supplementary Figure 1 MTDR potently inhibits the mitochondrial oxygen consumption rate in MDA-MB-468 cells. A representative Seahorse tracing is shown, with bar graphs highlighting the quantitative, dose-dependent effects of MTDR on basal respiration, maximal respiration and ATP production. Note that MTDR treatment exhibits near complete inhibition of OCR in MDA-MB-468 cells at 500 nM. The statistical test used was an unpaired one-tail t-test.

Supplementary Figure 2 MTDR has no effect on glycolysis in MDA-MB-468 cells. A representative Seahorse tracing is shown, with bar graphs highlighting the quantitative, dose-dependent effects of MTDR on glycolysis, glycolytic capacity and glycolytic reserve. Note that MTDR has no significant effect on glycolysis, at concentrations up to 1 μM. The statistical test used was an unpaired one-tail t-test.

Supplementary Figure 3 HITC inhibits mitochondria aerobic respiration. Adherent MCF7 cells were treated with HITC using five different concentrations for 16 hours. After treatment, the mitochondrial oxygen consumption rate (OCR) was measured using the Seahorse XFe96 analyzer. Data is expressed as percentage of OCR vs control. All data was normalized for cell number. Statistical analysis was conducted using one-way ANOVA.

Supplementary Figure 4 HITC induces glycolysis. Adherent MCF7 cells were treated with HITC using five different concentrations for 16 hours. After treatment, the extracellular acidification rate (ECAR) was measured using the Seahorse XFe96 analyzer. Data is expressed as percentage of ECAR vs control. All data was normalized for cell number. Statistical analysis was conducted using one-way ANOVA.

Supplementary Figure 5 DDI does not inhibit mitochondria aerobic respiration. Adherent MCF7 cells were treated with DDI using five different concentrations for 16 hours and the mitochondrial OCR was measured using the Seahorse XFe96 analyzer. Data is expressed as percentage of OCR vs control. Statistical analysis was conducted using one-way ANOVA.

Supplementary Figure 6 DDI does not induce glycolysis. Adherent MCF7 cells were treated with DDI using five different treatment concentrations, the ECAR was measured using the Seahorse XFe96 analyzed. Data is expressed as percentage of ECAR vs control. Statistical analysis was conducted using one-way ANOVA.

Supplementary Figure 7 Cyanine 5 analog uptake by MCF7 mammospheres. Microscopy analysis of MCF7 dye internalization at a concentration 50 nM for each analog was detected in the Cy5 fluorescent channel (red) and is visualized with nuclear staining detected in the DAPI channel (blue). Images were acquired with an EVOS fluorescent microscope, using Cy5 channel and a 20x objective.
