# Supplementary figures and images for: MitoTracker Deep Red (MTDR) Is a Metabolic Inhibitor for Targeting Mitochondria and Eradicating Cancer Stem Cells (CSCs), With Anti-Tumor and Anti-Metastatic Activity In Vivo
Source: Front Oncol. 2021 Jul 30;11:678343. doi: 10.3389/fonc.2021.678343 (PMC8361836; doi:10.3389/fonc.2021.678343)

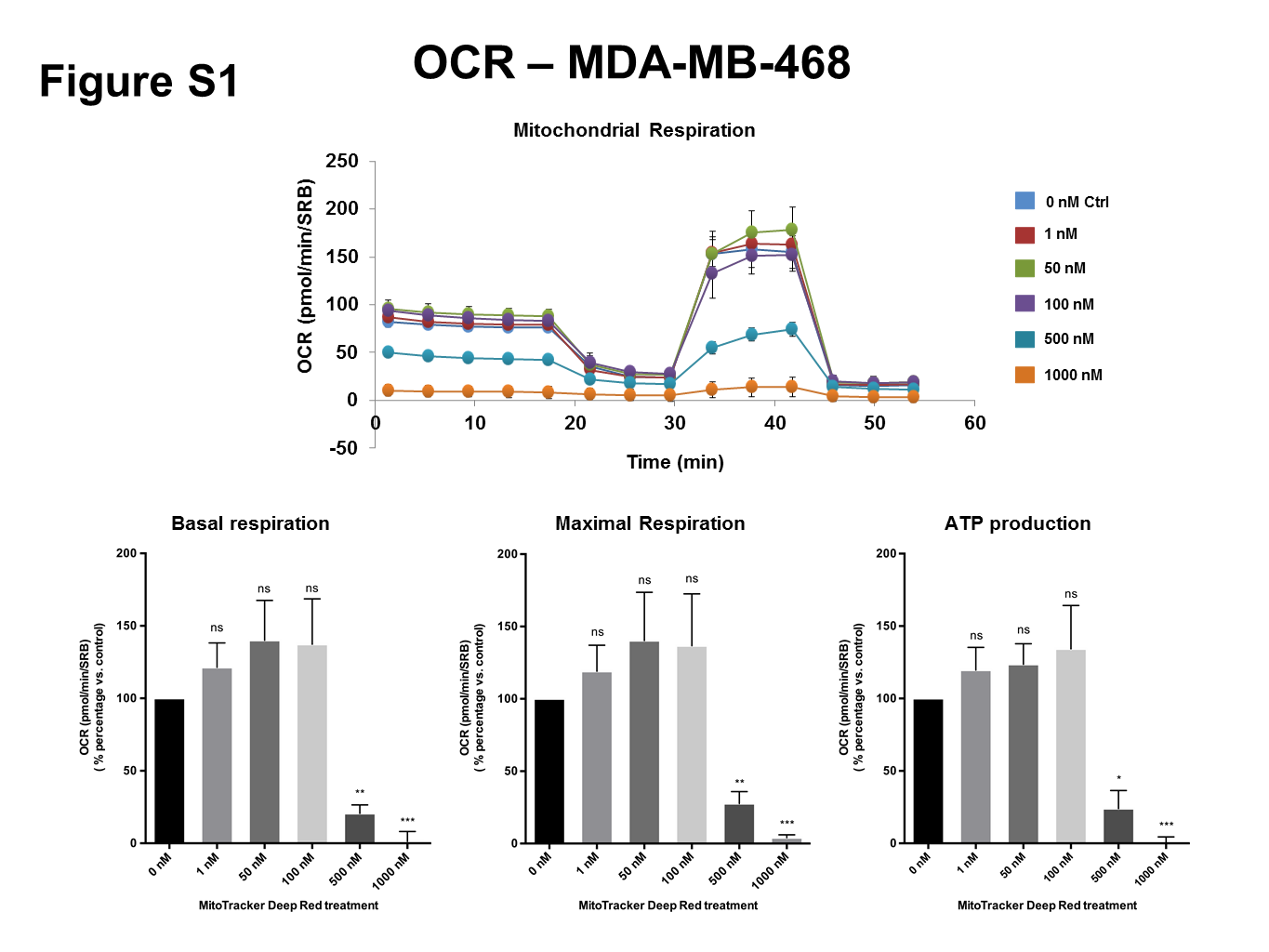

Supplement: Supplementary Figure 1 — MTDR potently inhibits the mitochondrial oxygen consumption rate in MDA-MB-468 cells. A representative Seahorse tracing is shown, with bar graphs highlighting the quantitative, dose-dependent effects of MTDR on basal respiration, maximal respiration and ATP production. Note that MTDR treatment exhibits near complete inhibition of OCR in MDA-MB-468 cells at 500 nM. The statistical test used was an unpaired one-tail t-test. [file Image_1.tif]

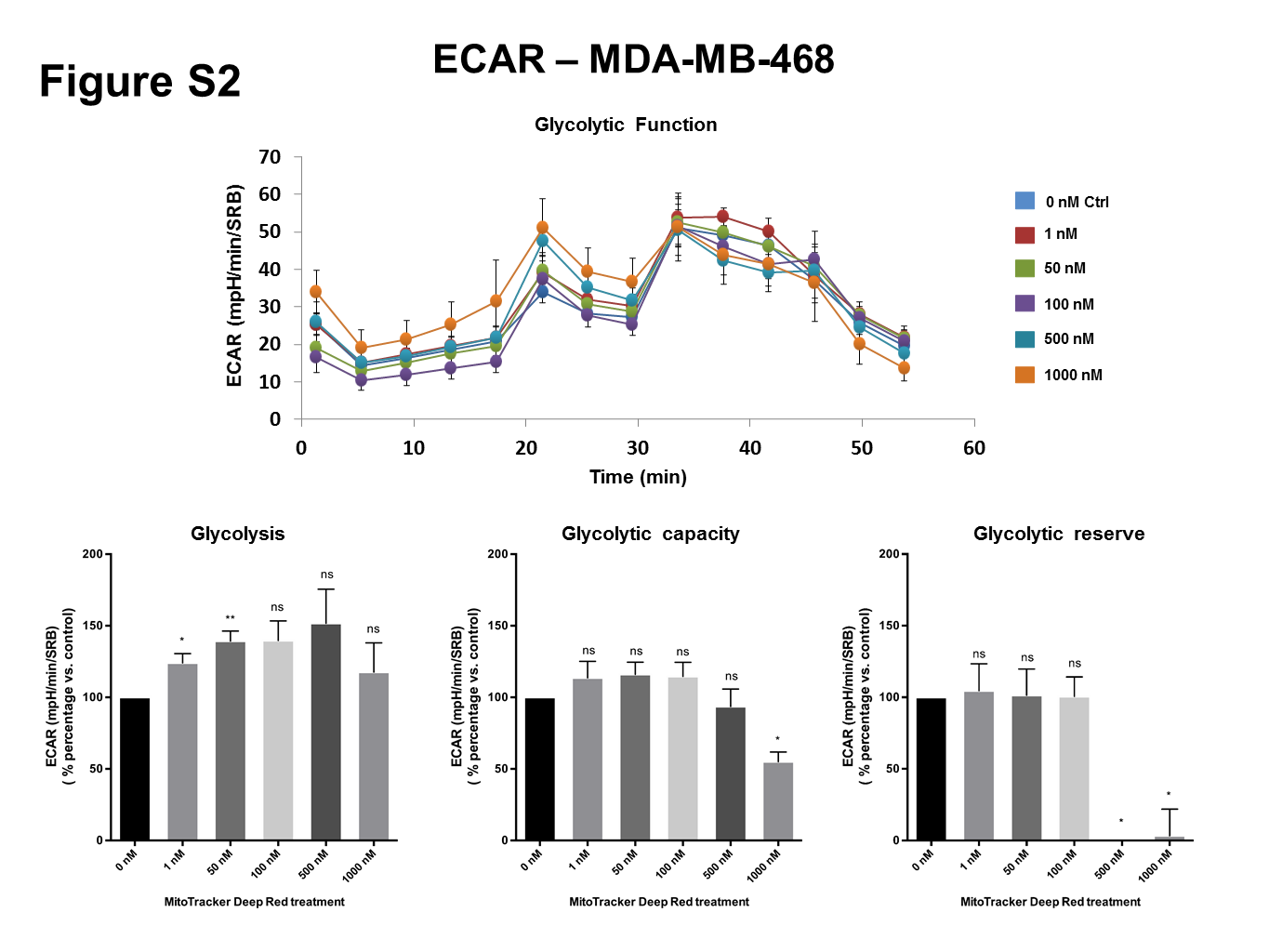

Supplement: Supplementary Figure 2 — MTDR has no effect on glycolysis in MDA-MB-468 cells. A representative Seahorse tracing is shown, with bar graphs highlighting the quantitative, dose-dependent effects of MTDR on glycolysis, glycolytic capacity and glycolytic reserve. Note that MTDR has no significant effect on glycolysis, at concentrations up to 1 μM. The statistical test used was an unpaired one-tail t-test. [file Image_2.tif]

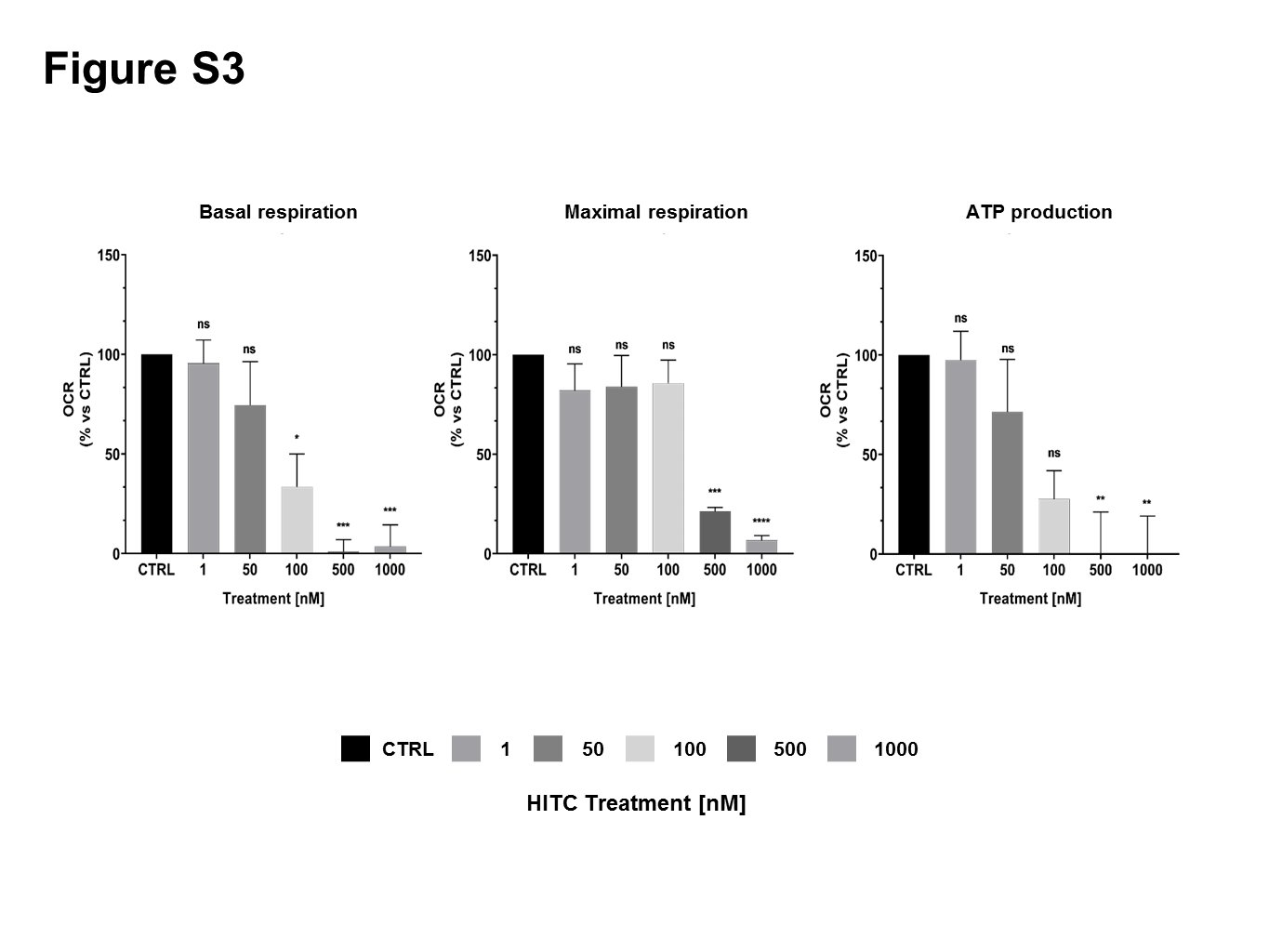

Supplement: Supplementary Figure 3 — HITC inhibits mitochondria aerobic respiration. Adherent MCF7 cells were treated with HITC using five different concentrations for 16 hours. After treatment, the mitochondrial oxygen consumption rate (OCR) was measured using the Seahorse XFe96 analyzer. Data is expressed as percentage of OCR vs control. All data was normalized for cell number. Statistical analysis was conducted using one-way ANOVA. [file Image_3.tif]

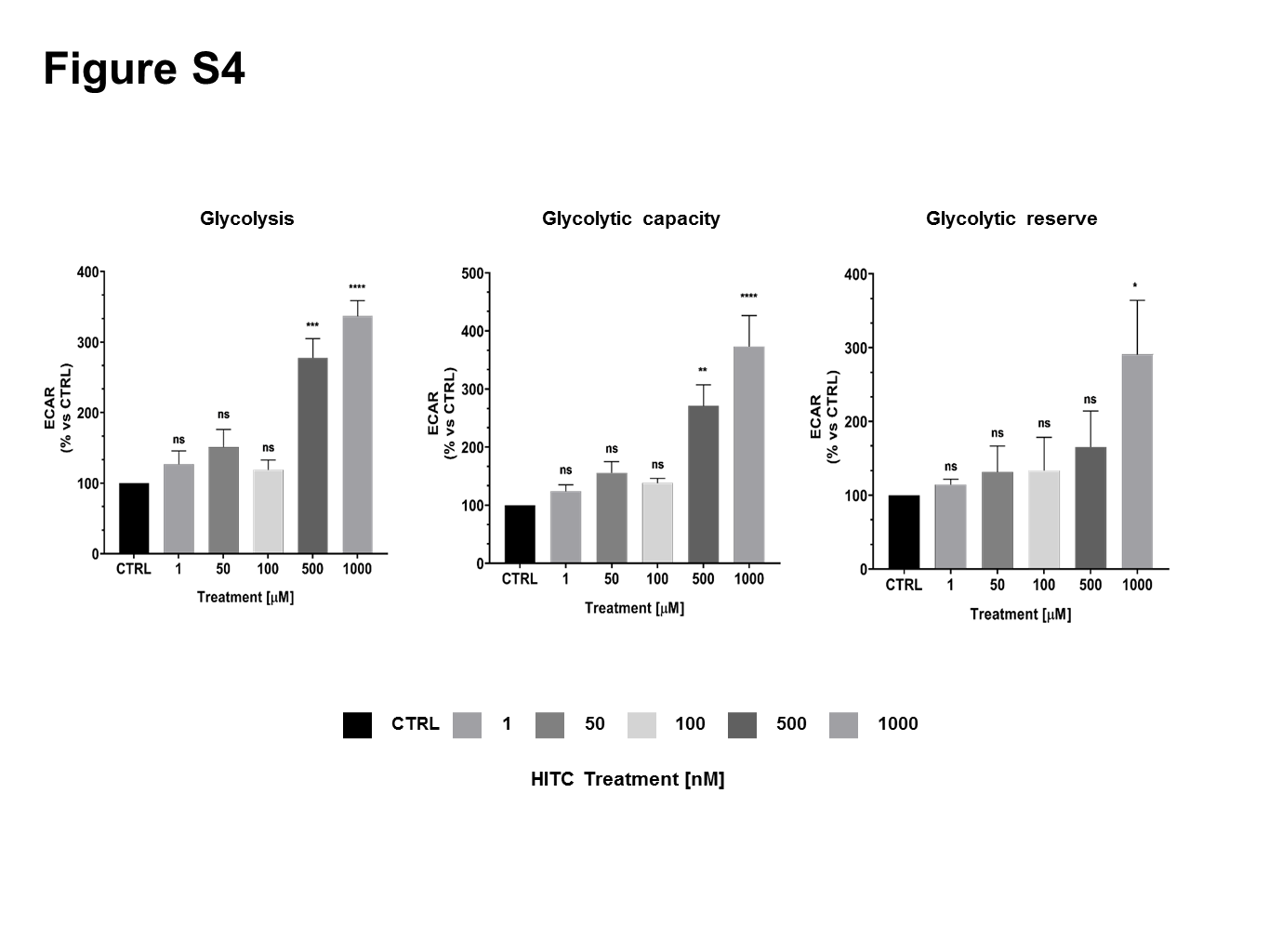

Supplement: Supplementary Figure 4 — HITC induces glycolysis. Adherent MCF7 cells were treated with HITC using five different concentrations for 16 hours. After treatment, the extracellular acidification rate (ECAR) was measured using the Seahorse XFe96 analyzer. Data is expressed as percentage of ECAR vs control. All data was normalized for cell number. Statistical analysis was conducted using one-way ANOVA. [file Image_4.tif]

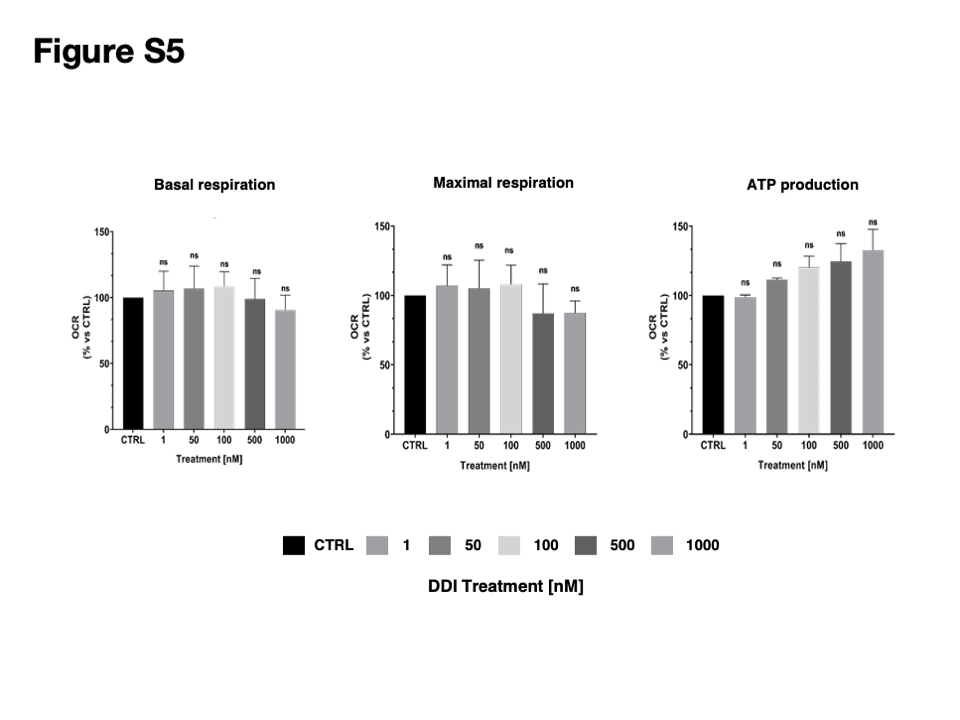

Supplement: Supplementary Figure 5 — DDI does not inhibit mitochondria aerobic respiration. Adherent MCF7 cells were treated with DDI using five different concentrations for 16 hours and the mitochondrial OCR was measured using the Seahorse XFe96 analyzer. Data is expressed as percentage of OCR vs control. Statistical analysis was conducted using one-way ANOVA. [file Image_5.tiff]

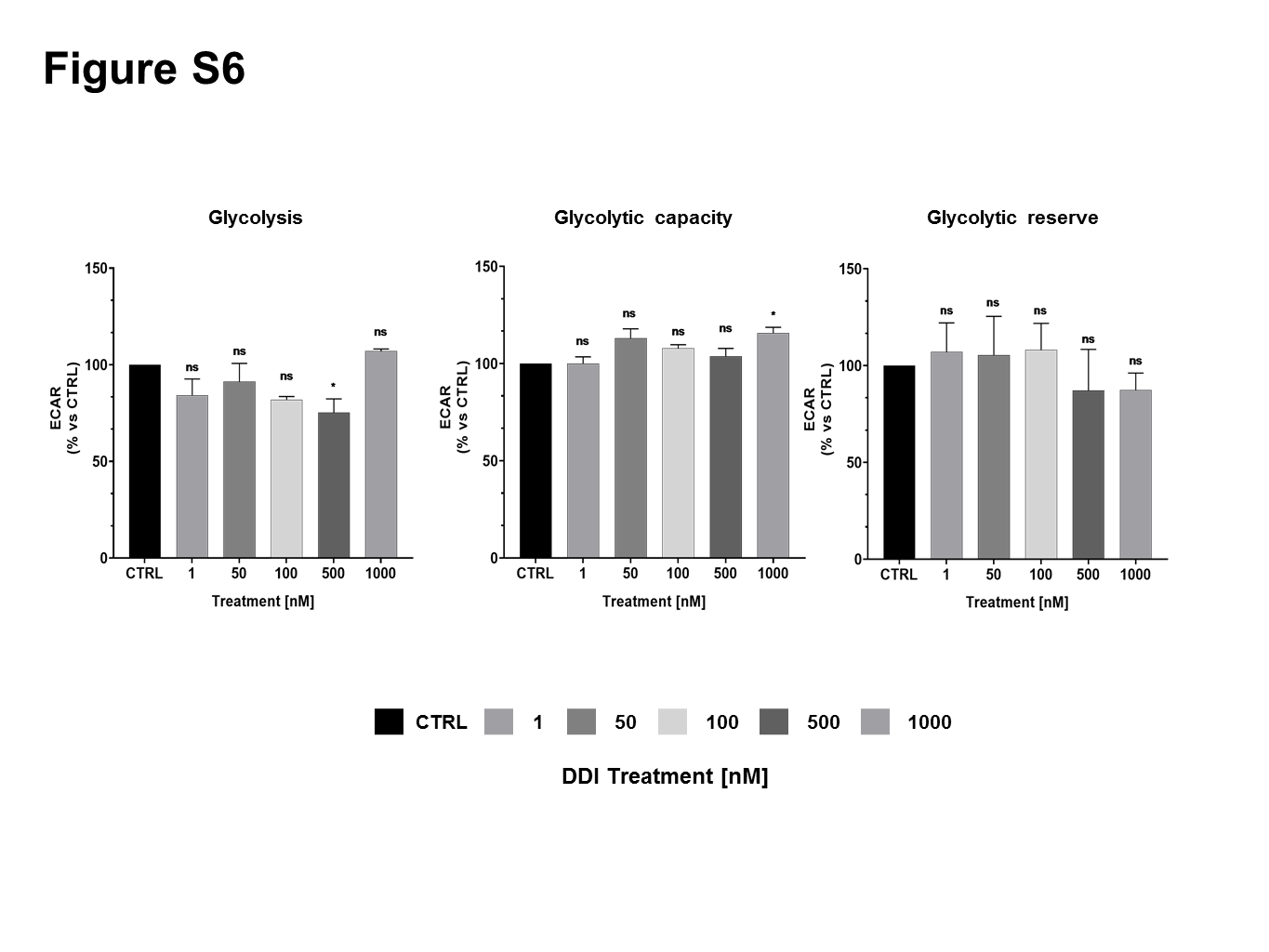

Supplement: Supplementary Figure 6 — DDI does not induce glycolysis. Adherent MCF7 cells were treated with DDI using five different treatment concentrations, the ECAR was measured using the Seahorse XFe96 analyzed. Data is expressed as percentage of ECAR vs control. Statistical analysis was conducted using one-way ANOVA. [file Image_6.tif]

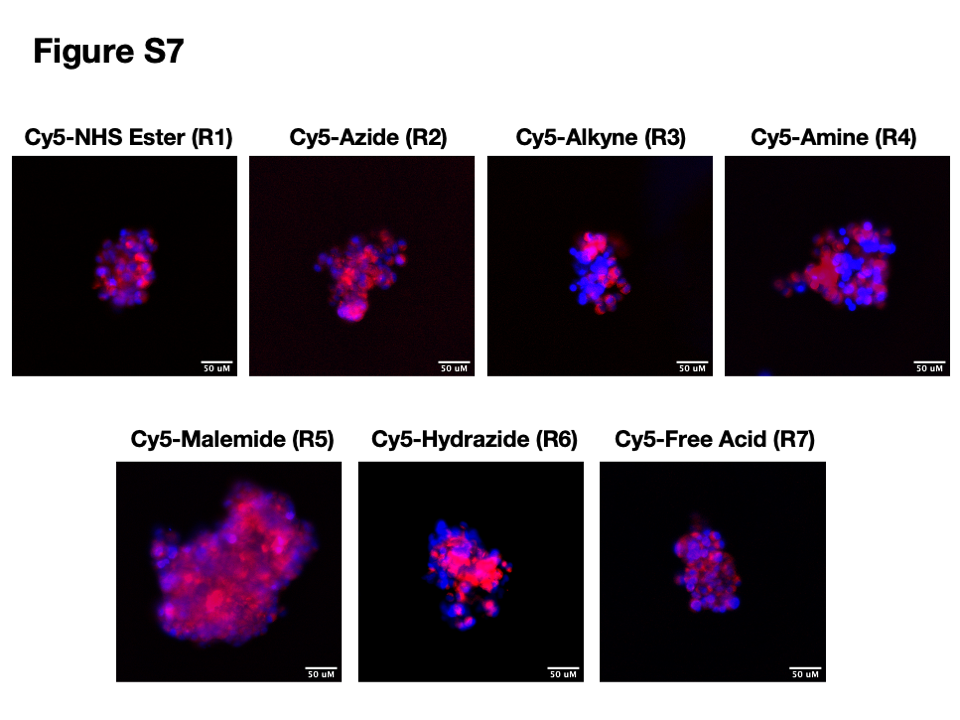

Supplement: Supplementary Figure 7 — Cyanine 5 analog uptake by MCF7 mammospheres. Microscopy analysis of MCF7 dye internalization at a concentration 50 nM for each analog was detected in the Cy5 fluorescent channel (red) and is visualized with nuclear staining detected in the DAPI channel (blue). Images were acquired with an EVOS fluorescent microscope, using Cy5 channel and a 20x objective. [file Image_7.tiff]
